# Gender and Psychological State, More than Pandemic-Related Lifestyle Change, Are Associated with Emotional Over-Eating Among University Students and Staff in Moldova

**DOI:** 10.3390/nu18213688

**Published:** 2026-11-07

**Authors:** Rodica Siminiuc, Dinu Țurcanu, Sergiu Siminiuc

**Affiliations:** 1 Faculty of Food Technology, Technical University of Moldova, MD-2004 Chișinău, Moldova; dinu.turcanu@utm.md; 2 Faculty of Computers, Informatics and Microelectronics, Technical University of Moldova, MD-2004 Chișinău, Moldova; sergiu.siminiuc@doctorat.utm.md

**Keywords:** appetitive traits, Adult Eating Behaviour Questionnaire, emotional over-eating, physical activity, self-rated health, mental health, COVID-19, university population, Republic of Moldova

## Abstract

**Background/Objectives**: Appetitive traits are rarely studied in Eastern Europe, and few pandemic surveys measured individual lifestyle change. This study tested whether retrospectively reported pandemic-related changes in physical activity and perceived health are associated with emotional over-eating independently of gender, cohort and psychological state, and preliminarily evaluated the Adult Eating Behaviour Questionnaire (AEBQ) in Romanian. **Methods**: Cross-sectional online survey at the Technical University of Moldova (2021; 623 students, 121 staff) with the AEBQ, pre-pandemic and current physical activity, and health items derived from the 36-item Short Form Health Survey, analysed by confirmatory factor analysis, invariance testing, multivariable regression and equivalence tests. **Results**: The eight-factor model fitted acceptably with diagonally weighted least squares (comparative fit index 0.911, root mean square error of approximation 0.057) and met change-in-fit criteria for scalar invariance across gender; Cronbach’s *α* ranged from 0.63 to 0.91. Reported median moderate-to-vigorous activity fell by more than half in students and by 44% in staff (*p* < 0.001); 37% of staff and 22% of students reported worse health than before the pandemic. In the primary linear model, emotional over-eating was associated with female gender, more emotional role limitations, a lower mental health score and lower current activity, whereas the reported decline in activity, perceived health deterioration and confirmed infection were not; equivalence tests excluded lifestyle-change effects beyond ±0.10 scale points. The secondary logistic model was consistent except for current activity. **Conclusions**: Emotional over-eating was associated with gender and psychological state more than with reported pandemic-related lifestyle change; if confirmed prospectively, this would support integrating mental-health support into university nutrition services. The Romanian AEBQ provided internally consistent emotional-eating subscales.

## 1. Introduction

The COVID-19 pandemic disrupted dietary habits, physical activity and mental health on a global scale. Studies across Europe, Asia and the Americas documented increases in emotional eating, snacking and irregular meal timing during lockdown periods, with unhealthy dietary changes reported by 30–50% of respondents in some populations [1,2,3]. Disruption of social eating, reduced opportunities for physical activity, and increased psychological strain were documented in adult and adolescent populations alike [4,5,6,7,8]. Meta-analytic syntheses across both pandemic waves confirm a small but consistent rise in emotional eating in the general population [9].

Physical activity declined substantially, as shown by both device-based and self-report data [10,11], and mental health deteriorated: surveys of higher-education students and systematic reviews of the general population reported a marked rise in anxiety and depressive symptoms [12,13], and in student cohorts followed longitudinally, symptoms had not returned to pre-pandemic levels two years later [14]. Psychological distress is an established trigger of emotional eating, and physical activity is a recognised modulator of appetite regulation [15,16,17,18]. Women report more emotional eating than men, a gender difference that persisted during the pandemic and was most pronounced among young adults [19]. It remains unclear, however, whether emotional eating during the pandemic tracked the changes in lifestyle that the pandemic imposed, namely the loss of physical activity, the deterioration of perceived health and infection itself, or whether it followed the individual’s current psychological state and gender, regardless of how that state arose. Few surveys could address this question, because most measured lifestyle at one point in time, without any pre-pandemic reference for the same individuals.

The Behavioural Susceptibility Theory of obesity posits that individual differences in appetitive traits, such as food responsiveness, emotional eating and satiety sensitivity, interact with environmental food cues to determine energy intake [20,21]. Appetitive traits are therefore the appropriate level at which to examine whether a lifestyle disruption of the pandemic’s magnitude is related to eating behaviour.

The Adult Eating Behaviour Questionnaire (AEBQ), the adult counterpart of the Child Eating Behaviour Questionnaire [22], measures eight such traits: the food-approach dimensions, Enjoyment of Food (EF), Emotional Over-Eating (EOE), Food Responsiveness (FR) and Hunger (H), and the food-avoidance dimensions, Emotional Under-Eating (EUE), Food Fussiness (FF), Slowness in Eating (SE) and Satiety Responsiveness (SR). The instrument has since been validated in several countries, including Australia, Norway, Mexico and China [23,24,25,26] and Italy [27]; to our knowledge, no Romanian-language version has been evaluated, although Romanian versions of two other emotional-eating instruments, the Dutch Eating Behavior Questionnaire and the Emotional Eater Questionnaire, have recently been validated [28,29], and Eastern European samples remain scarce.

Two further gaps concern the population and the measures. Pandemic research on universities has concentrated on students; academic staff, who differ from students in age, occupational demands and family responsibilities, have rarely been studied alongside them. Self-perceived health, a multidimensional construct encompassing physical functioning, pain, emotional well-being and social participation [30], has seldom been examined together with physical activity and appetitive traits in the same sample. The present study addresses these gaps in a university population of the Republic of Moldova, a Romanian-speaking country of Eastern Europe, a region in which pandemic-era food-related behaviour has so far been documented largely through online-media indicators [31] rather than with validated instruments.

The question addressed here is not confined to the pandemic period in which the data were collected. The pandemic constituted a large, population-wide perturbation of lifestyle and therefore a natural setting in which to test whether emotional over-eating is associated with lifestyle change or with the individual’s psychological state, a distinction that bears on how emotional eating is understood and addressed in any period of disruption. Appetitive-trait data collected during the pandemic together with a within-person pre-pandemic reference can no longer be collected. The persistence of the mental-health effects documented above gives the psychological correlates identified here continuing relevance in the post-pandemic period.

The primary aim was to test whether pandemic-related lifestyle change is associated with emotional over-eating independently of gender, cohort and psychological state. Four specific objectives served this aim:

(1) To quantify pandemic-related change in physical activity and self-rated health at the individual level, using retrospective pre-pandemic reports, among students and academic staff of the Technical University of Moldova (TUM);
(2) To provide a first evaluation of the reliability and factor structure of the AEBQ in Romanian;
(3) To compare appetitive-trait profiles, including a combined emotional-eating profile, between cohorts and genders;
(4) To examine the correlates of appetitive traits.

The central question was whether the individual-level pandemic changes retain an independent association with emotional over-eating once gender, cohort and psychological state are accounted for.

## 2. Materials and Methods

### 2.1. Study Design and Setting

This was a cross-sectional observational study conducted at TUM, Chișinău, Republic of Moldova, in October–November 2021, when the university operated under a hybrid model of partial in-person and partial online instruction. Data were collected with two parallel online questionnaires, one for students and one for academic staff, built in Google Forms (Google LLC, Mountain View, CA, USA) and distributed through institutional channels. Sampling was by convenience: all students and staff could participate, no eligibility criteria beyond TUM affiliation and adult age were applied, and the number of persons reached by the invitation was not recorded, so a participation rate cannot be computed. For physical activity and general health, respondents reported on two reference periods, the period before the pandemic (early 2020) and the current pandemic period; these retrospective paired reports are used to characterise pandemic-related change at the individual level.

### 2.2. Participants

The final sample comprised 623 students and 121 academic staff who submitted the questionnaire; no respondent selected the under-18 age category. Individual items could be left unanswered, so the number of valid responses varied across instruments; the effective *n* is therefore reported for every analysis, and the flow of valid responses per instrument is given in Table S1.

### 2.3. Instruments

Block 1 (Demographics) collected self-reported gender (a single item with the options female and male; the term gender is used throughout to denote this self-report), age group and self-reported COVID-19 history (confirmed, not confirmed, unknown).

Block 2 (Physical Activity) was adapted from the Global Physical Activity Questionnaire (GPAQ) [32] and assessed three intensity levels (vigorous, moderate and light), each with frequency (days/week) and duration (hours/day), reported separately for the pre-pandemic and the current pandemic period. Duration was answered in categories and converted to hours as follows: less than one hour = 0.5 h, one to eight hours = the stated value, more than eight hours = 9 h. Weekly energy expenditure was expressed in metabolic equivalent of task minutes per week (MET-min/week), obtained, as in GPAQ, from vigorous and moderate activity only, as the product of the intensity-specific MET value, the number of active days per week and the daily duration in minutes, summed over the two levels:

(1) $$\mathrm{MET}\text{-}\min/\mathrm{week}\;=\;8.0\;\times \;d_{\mathrm{vig}}\;\times \;t_{\mathrm{vig}}\;+\;4.0\;\times \;d_{\mathrm{mod}}\;\times \;t_{\mathrm{mod}}$$

where *d* and *t* denote the frequency (days/week) and the daily duration (min/day) of activity, the subscripts vig and mod refer to vigorous and moderate activity, and 8.0 and 4.0 are the corresponding MET values. Light activity was recorded for descriptive purposes but not scored, in accordance with the GPAQ protocol. Respondents were classified according to the GPAQ analysis guide [33] as inactive (<600 MET-min/week), minimally active (600–3000) or health-enhancing physically active (>3000); the 600 MET-min/week threshold corresponds to the World Health Organization (WHO) recommendation of 150 min of moderate-intensity activity per week [34]. Following the same guide, reports totalling more than 16 h of activity per day were treated as implausible and set to missing. Pandemic-related change in activity was computed as pandemic minus pre-pandemic MET-min/week, both obtained from Equation (1). Converting duration categories to fixed values introduces a rounding error of up to ±0.5 h/day within each category and of unknown size above eight hours; this error is unlikely to depend on eating behaviour and therefore adds imprecision to the MET-min/week estimates and to the change scores and may attenuate their associations with eating behaviour. Absolute MET-min/week values should therefore be read as approximate; the activity categories are affected only for respondents close to the 600 and 3000 MET-min/week thresholds.

Block 3 (Self-Perceived Health) was adapted from the 36-item Short Form Health Survey (SF-36) [30] and included: a general health self-rating for the current period (1 = poor to 5 = excellent); a single retrospective item comparing current health with the pre-pandemic period (1 = much worse to 5 = much better, 3 = the same), hereafter the health-comparison item; role limitations due to physical health (4 yes/no items, summed 0–4); role limitations due to emotional problems (3 yes/no items, summed 0–3); interference of health with social activities (one item, 1 = not at all to 5 = extremely); bodily pain in the past four weeks (yes/no) and its interference with usual activities (same 5-point scale); a mental health composite of eight items from the SF-36 vitality and mental health scales (6-point frequency scale, negative items reverse-scored, averaged so that higher scores indicate better mental health); and general health perceptions (4 items, 1 = definitely false to 5 = definitely true, negative items reverse-scored, averaged). The items were drawn from the corresponding SF-36 scales and retain the original response formats; because selected items rather than complete scales were administered, the resulting measures are study-specific, SF-36-derived indicators (hereafter the mental health score, the emotional role limitations count and the health-perception score), covering the domains examined here: psychological state, role functioning and perceived health. Limitation counts and pain presence were set to missing when the whole block was left blank; unanswered items within an answered block were counted as not endorsed, so that respondents with partially completed blocks were retained.

Block 4 (Eating Behaviour) used the AEBQ [22]: 35 items in eight subscales, rated on a 5-point Likert scale (1 = strongly disagree to 5 = strongly agree). Reverse-scored items (three Food Fussiness items and the Slowness in Eating item “I often finish my meals quickly”) were recoded as 6 − score. The Romanian version was produced by the research team through direct translation of the English items; no formal back-translation, expert-panel review or pilot testing was carried out. The psychometric analyses reported here therefore constitute a preliminary evaluation of the Romanian AEBQ. During the present analysis, the item intended as “I eat less when I’m irritable” was found to have been rendered with the same Romanian wording as “I eat less when I’m annoyed” (“Mănânc mai puțin când sunt enervat”); the duplicate was excluded, and Emotional Under-Eating was scored on its four unique items. Subscale scores were computed as the mean of the constituent items (range 1–5) when at least 75% of the items had been answered.

### 2.4. Statistical Analysis

All analyses were performed in Python 3.11–3.13 (pandas 3.0.2, SciPy 1.17.1, statsmodels 0.15.0, pingouin 0.6.1, semopy 2.3.11) and, for measurement invariance, in R 4.5.3 (lavaan 0.7-2, semTools 0.5.9) [35]. Missing responses were handled by pairwise deletion, and the effective *n* is reported for every estimate. Categorical variables are summarised as *n* (%), subscale and health scores as mean ± standard deviation (SD), and physical activity, whose distribution was strongly right-skewed, as median [interquartile range, IQR]. Internal consistency was assessed with Cronbach’s *α* and Feldt 95% confidence intervals (CI) per subscale, cohort and gender, and for the two SF-36-derived composite scores, complemented by item–rest correlations. The eight-factor structure of the AEBQ was tested by confirmatory factor analysis on the 34 retained items with diagonally weighted least squares (DWLS), appropriate for ordinal indicators, with maximum-likelihood estimates reported for reference; model fit was judged against a comparative fit index (CFI) and Tucker–Lewis index (TLI) ≥ 0.90 and a root mean square error of approximation (RMSEA) ≤ 0.06 [36]. Measurement invariance of the eight-factor model across gender and across cohort was then tested in the combined sample with complete AEBQ data. For gender, the items were treated as ordinal, with weighted least squares mean- and variance-adjusted (WLSMV) estimation and delta parameterisation, and the configural, threshold, metric (thresholds and loadings) and scalar (thresholds, loadings and intercepts) models were fitted in sequence under the identification constraints of Wu and Estabrook [37]. Because one response category was unobserved in the staff group, the cohort comparison and a reference analysis for gender treated the items as continuous with robust maximum likelihood (MLR), comparing the configural, metric and scalar models. Invariance at each step was accepted when ΔCFI ≥ −0.010 and ΔRMSEA ≤ 0.015 [38]; scaled χ^2^ difference tests are also reported. Subscale scores are derived from ordinal items and skewed (Shapiro–Wilk *p* < 0.001 for all subscales), so non-parametric methods were used for group comparisons. Gender and cohort differences were tested with the Mann–Whitney *U* test, with the effect size

(2) $$r=\frac{Z}{\sqrt{N}}$$

where *Z* is the standardised test statistic, and *N* is the number of observations in the comparison; differences by COVID-19 status and physical activity category were tested with the Kruskal–Wallis *H* test, with the effect size

(3) $$\eta^{2}=\frac{H-k+1}{n-k}$$

where *k* is the number of groups and *n* is the number of observations. A Bonferroni-corrected threshold was applied across the eight subscales (*p* < 0.00625). Within-person change in physical activity was tested with the Wilcoxon signed-rank test, with *r* computed as in Equation (2) from the number of pairs; distributions of the health-comparison item and of emotional-eating profiles across groups were compared with *χ*^2^ tests and Cramér’s *V*. Emotional-eating profiles were defined from the agreement side of the response scale (subscale score ≥ 3): over-eating only, under-eating only, both or neither. Spearman’s *ρ* assessed bivariate associations between subscale scores and physical activity, health and mental-health variables; these correlations are exploratory and are interpreted by effect magnitude; unadjusted *p* values are reported in the main text, and Benjamini–Hochberg false discovery rate (FDR)-adjusted *q* values [39], computed within each family of tests (the correlate × subscale matrix per cohort; the subscale intercorrelations per cohort and in the combined sample) with *q* < 0.05 as the criterion, are reported in the Supplementary Materials.

Predictors of EOE were examined by ordinary least squares regression with heteroscedasticity-consistent (HC3) standard errors and, as a secondary analysis, for high EOE (score ≥ 3.5, corresponding to the sample mean plus one SD, 2.56 + 0.95, and lying above the neutral midpoint of the response scale) by binary logistic regression with odds ratios (OR) and 95% confidence intervals; the same models were fitted for SR and H as supplementary analyses. The primary model (Model 1) included gender, cohort, confirmed COVID-19 infection, current physical activity (log-transformed) and change in physical activity, both standardised on the full sample, the health-comparison item, the mental health score and emotional role limitations; age group, whose categories differ between the two questionnaires and which is collinear with the cohort, was examined separately: cohort differences in subscale scores were re-estimated in the age-overlapping subgroup (students aged 24 years or older and staff aged 30 years or younger) with the Mann–Whitney *U* test and with Model 1 refitted in this subgroup, and age gradients within each cohort were tested across age groups with the Kruskal–Wallis *H* test and Spearman’s *ρ*. Sensitivity analyses added current self-rated health (Model 1b; missing for 11% of respondents) and the EF and EUE subscales (Model 2), repeated the logistic model with alternative thresholds for high EOE (score ≥ 3.0 and ≥4.0), and re-estimated Model 1 with the emotional role limitations count scored from complete item sets only and, alternatively, prorated to the number of answered items. Models were fitted on complete cases, and multicollinearity was checked with variance inflation factors (all <1.5). Because the primary hypothesis concerned the absence of an effect, the Model 1 coefficients of the three pandemic-related variables (change in physical activity, the health-comparison item and confirmed infection) were also subjected to two one-sided tests of equivalence (TOST) [40] with the HC3 standard errors, using ±0.10 EOE scale points per unit of the predictor as the smallest effect of interest; this margin equals about one-tenth of the EOE standard deviation (0.95) and 2.5% of the 1–5 scale range, a difference at the lower bound of what is conventionally regarded as a small effect, and margins of ±0.05, ±0.15 and ±0.20 were examined in sensitivity analyses. Equivalence is concluded when the 90% confidence interval lies entirely within the margin. No a priori sample-size calculation was performed. Significance was set at *p* < 0.05 (two-tailed) unless otherwise stated.

### 2.5. Ethical Considerations

The study was conducted in accordance with the Declaration of Helsinki. Participation was voluntary and fully anonymous; no personal identifying data were collected. Before accessing the questionnaire, participants read an information page describing the study purpose, the voluntary nature of participation and the absence of personal data collection, and provided informed digital consent by proceeding to the questionnaire. Under the institutional regulations of the Technical University of Moldova applicable to non-interventional, anonymous studies of adult volunteers, the study did not require prior formal approval by an ethics committee; the Ethics Committee of the Faculty of Food Technology confirmed this in a written statement and attested that the study complied with institutional and international ethical standards (Ethics Committee Statement No. 6, April 2026).

## 3. Results

### 3.1. Sample Characteristics

The combined sample comprised 744 participants: 623 students (83.7%) and 121 academic staff (16.3%). Their demographic profile, COVID-19 history, physical activity during the pandemic period and self-perceived health are summarised by cohort and gender, with the number of valid responses for each measure (Table 1).

The cohorts differed in age structure, gender composition, and COVID-19 exposure. The student cohort was predominantly male (58.1%) and concentrated in the 18–23 age range (83.6%), whereas the staff cohort was predominantly female (65.3%) and middle-aged, with 57.9% of respondents aged 31–50 years. Confirmed infection was reported by nearly half of staff (47.1%) but by less than a third of students (30.8%), whereas uncertainty about infection status was twice as prevalent among students (24.4% compared with 12.4%).

During the pandemic period, 29.7% of students and 25.9% of staff met the health-enhancing threshold, with median moderate-to-vigorous activity of 1440 and 1200 MET-min/week. Inactivity was more prevalent among women than among men in students (37.4% compared with 25.5%), whereas the gender difference among staff was negligible (36.1% compared with 33.3%).

Current self-rated health was moderate in both cohorts (means of 3.07 and 2.84 on the 1–5 scale). In both cohorts, men reported higher mental health scores and fewer emotional role limitations than women, a gender gap of similar size in students and staff.

### 3.2. Pandemic-Related Change in Physical Activity and Self-Rated Health

Respondents reported physical activity for both the pre-pandemic period (retrospectively) and the pandemic period, which allowed within-person reported change to be quantified for the 524 students and 103 staff with valid paired data. Paired distributions were compared with the Wilcoxon signed-rank test and the shift between GPAQ activity categories was tabulated (Table 2).

**Figure 1:**
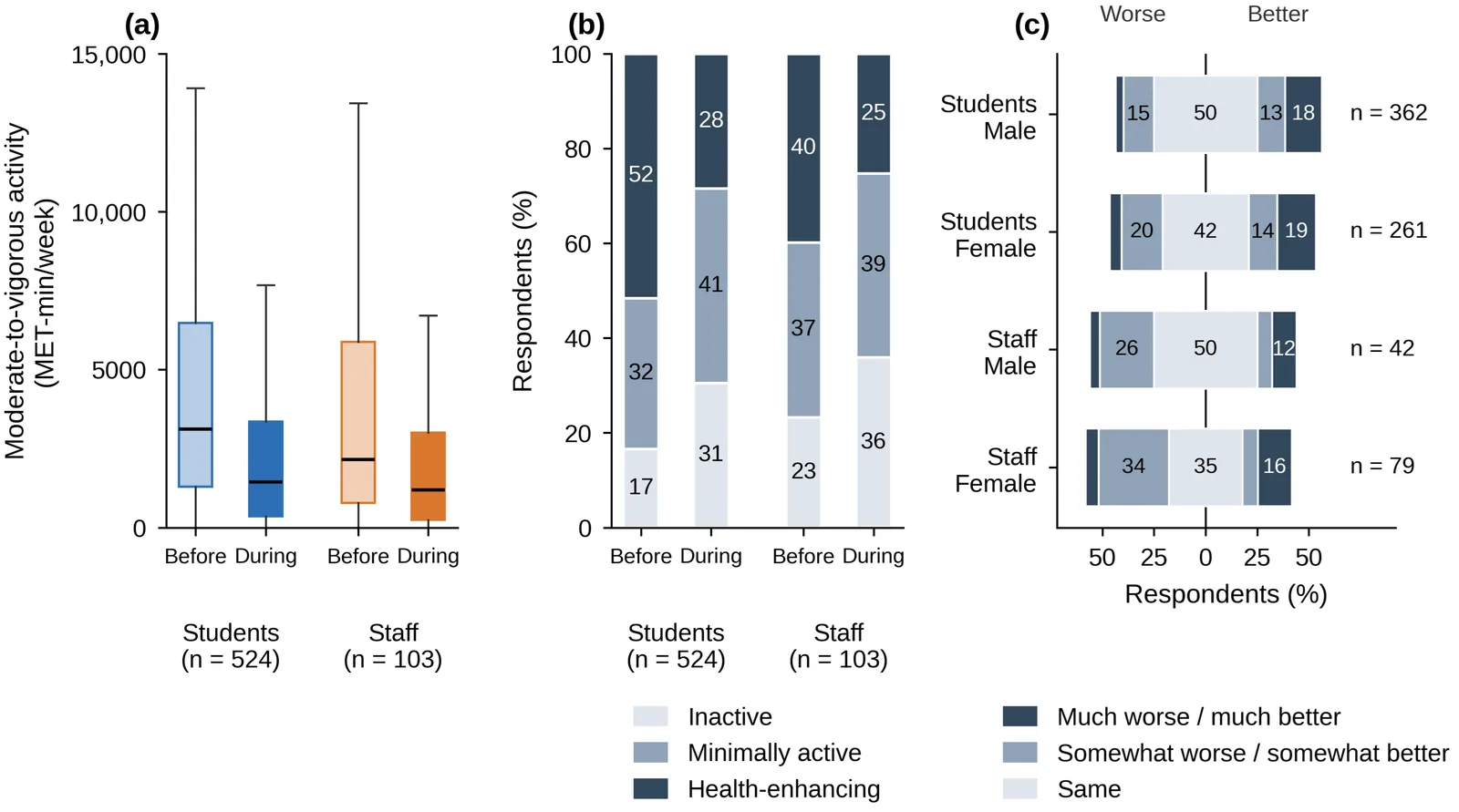
Pandemic-related change in physical activity and self-rated health by cohort and gender. (**a**) Moderate-to-vigorous physical activity before (light tint) and during (full colour) the pandemic in respondents with paired data; boxes show the median and interquartile range, whiskers extend to 1.5 × IQR; values beyond the whiskers are omitted for readability. (**b**) Distribution of GPAQ physical-activity categories before and during the pandemic (percentages within bars; segments below 5% unlabelled). (**c**) Self-rated health compared with the pre-pandemic period (health-comparison item) by cohort and gender; bars extend left of zero for worse and right of zero for better ratings, with darker shading for “much” and lighter for “somewhat”; the central light segment is “the same” (segments below 8% unlabelled). Blue: students; orange: staff (panel (**a**)); in panels (**b**,**c**), shading from light to dark encodes the ordered categories. IQR: interquartile range; MET: metabolic equivalent of task; GPAQ: Global Physical Activity Questionnaire.

Reported moderate-to-vigorous physical activity fell markedly in both cohorts: the median decreased from 3120 to 1440 MET-min/week in students and from 2160 to 1200 MET-min/week in staff, with 53.2% and 47.6% of respondents reporting lower values than before the pandemic (both *p* < 0.001, *r* = 0.502 in each cohort). Within the paired subsample, the share of respondents meeting the health-enhancing threshold declined from 51.5% to 28.4% in students and from 39.8% to 25.2% in staff, while the proportion classified as inactive rose from 16.6% to 30.5% and from 23.3% to 35.9%, respectively (Figure 1a,b).

Perceived health deteriorated more often among staff than among students: 37.2% of staff compared with 21.5% of students rated their current health as worse than before the pandemic, whereas improvement was reported by 31.8% of students and 22.3% of staff (χ^2^(2) = 14.21, *p* < 0.001, Cramér’s V = 0.138). Deterioration was reported more often by women than by men in both cohorts (students 25.7% compared with 18.5%; staff 40.5% compared with 31.0%), a difference that was nominally significant in students (χ^2^(2) = 6.06, *p* = 0.048, Cramér’s V = 0.099) but not in the smaller staff cohort (χ^2^(2) = 2.42, *p* = 0.299; Figure 1c). Full distributions of the health-comparison item are provided in Table S4.

### 3.3. Reliability and Factor Structure of the AEBQ

Prior to inferential analyses, the internal consistency of each AEBQ subscale was assessed with Cronbach’s *α*, computed separately for students, staff and the combined sample, and compared with the values reported in the original validation [22] (Table 3).

Internal consistency varied across subscales. EOE showed the highest reliability in both cohorts (*α* = 0.905 in students and 0.901 in staff), followed by EUE on its four unique items (*α* = 0.871 and 0.853) and SE (*α* = 0.813 and 0.769); EOE and EUE closely matched, and SE exceeded, the values of the original validation (0.89, 0.85 and 0.65). EF (*α* = 0.805) and H (*α* = 0.700) also met the conventional 0.70 threshold in students. By contrast, FF, FR and SR fell below it in students (*α* = 0.684, 0.662 and 0.641) and, with the exception of FR, in staff; results for these subscales are retained but interpreted with caution. Item–rest correlations for all 34 items and reliability by gender are given in Table S2.

The two SF-36-derived composite scores differed in reliability: the eight-item mental health score was highly consistent (*α* = 0.902 in students and 0.904 in staff), whereas the four-item health-perception score was only modestly so (*α* = 0.656 and 0.598).

The eight-factor measurement model was fitted to the 34 retained items by confirmatory factor analysis in the combined sample and, as a descriptive check of configural similarity, separately by cohort and by gender (Table 4).

With the DWLS estimator, the eight-factor model reached acceptable fit in the combined sample (CFI = 0.911, TLI = 0.900, RMSEA = 0.057; *n* = 621) and in students, men and women analysed separately (CFI 0.915–0.963, RMSEA 0.036–0.059); the maximum-likelihood solution, which treats the five-point items as continuous, fell below the conventional cut-offs (CFI = 0.863, RMSEA = 0.062). All standardised loadings were significant; the weakest belonged to the FF items “I often decide that I don’t like a food, before tasting it” (0.16) and “I refuse new foods at first” (0.27), consistent with the lower internal consistency of FF (Table S3). Fit was comparable in students, men and women; the staff-only solution is overfitted, and its indices do not inform model fit.

Measurement invariance was then tested in multigroup models. Across gender, with ordinal items, the configural model fell below the fit cut-offs (CFI = 0.896, RMSEA = 0.082; *n* = 621), and the successive constraints on thresholds, loadings and intercepts met the fit-index criteria at every step (ΔCFI = −0.002, −0.001 and −0.001; RMSEA did not increase), whereas the scaled χ^2^ difference tests were significant at each step (*p* < 0.001). Across cohort, with items treated as continuous, the metric model was supported by both criteria (ΔCFI = −0.001; Δχ^2^(26) = 30.6, *p* = 0.244) and the scalar model by the fit-index criteria (ΔCFI = −0.004, ΔRMSEA < 0.001) but not by the χ^2^ difference test (Δχ^2^(26) = 58.8, *p* < 0.001); the continuous-item reference models for gender gave the same pattern (ΔCFI = −0.005 and −0.007). The change-in-fit criteria for metric and scalar invariance were therefore met across gender and across cohort, although the χ^2^ tests indicate that some non-equivalence of item parameters cannot be excluded; full fit statistics are given in Table S7.

### 3.4. Appetitive-Trait Profiles by Cohort and Gender

**Figure 2:**
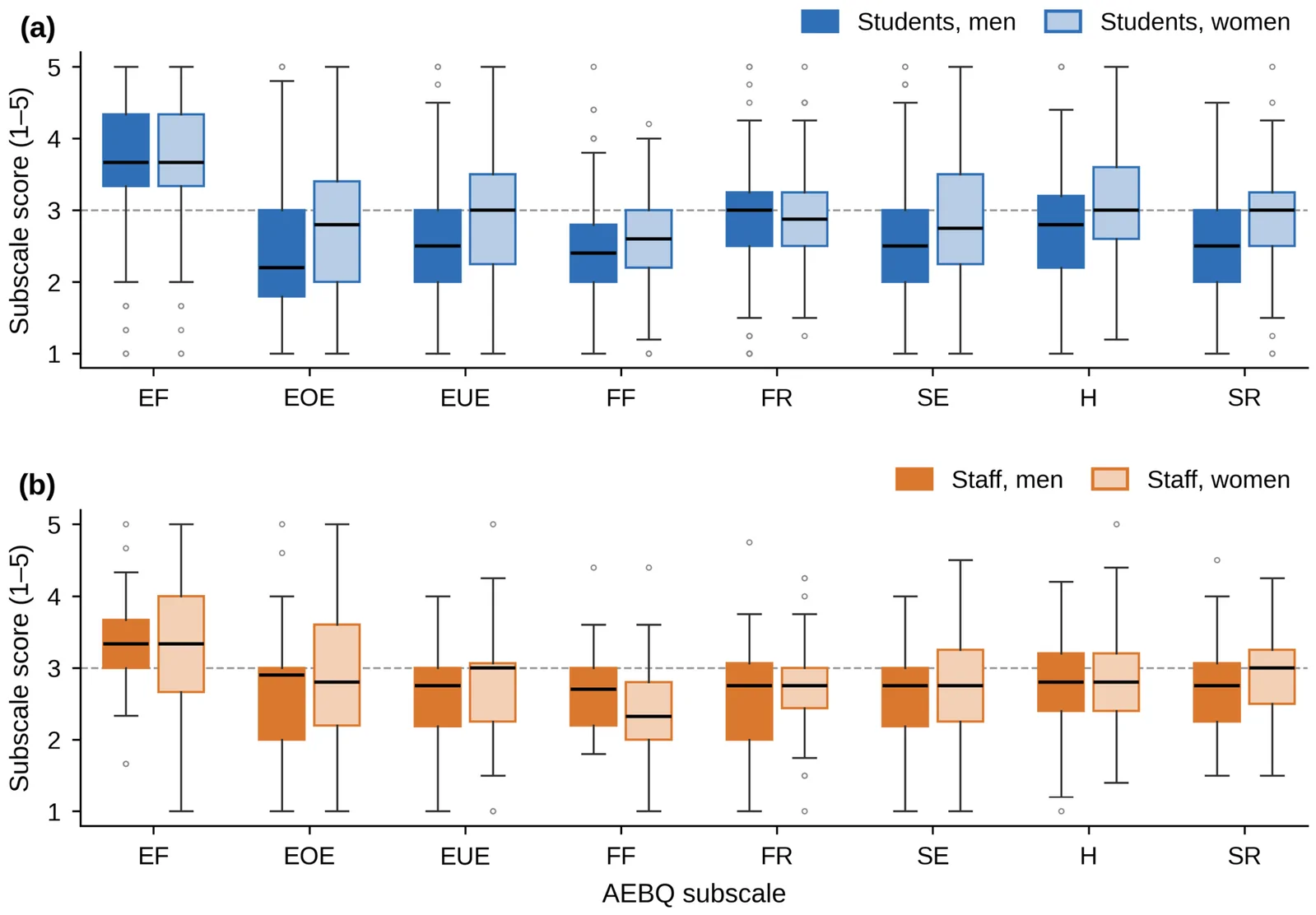
Distribution of AEBQ subscale scores by cohort and gender. (**a**) Students; (**b**) staff. Boxes show the median and interquartile range, whiskers extend to 1.5 × IQR and circles mark outliers. Blue: students; orange: staff; full colour: men; light tint: women. The dashed line marks the scale midpoint (3.0). AEBQ: Adult Eating Behaviour Questionnaire; EF: Enjoyment of Food; EOE: Emotional Over-Eating; EUE: Emotional Under-Eating; FF: Food Fussiness; FR: Food Responsiveness; SE: Slowness in Eating; H: Hunger; SR: Satiety Responsiveness; IQR: interquartile range.

Before testing group differences, the appetitive-trait profile of each cohort was characterised descriptively. The distribution of each subscale score was examined by cohort and gender, with the scale midpoint (3.0) as the reference (Figure 2).

EF was the highest-rated subscale in both cohorts (median 3.67 in students and 3.33 in staff) and the only trait with medians above the midpoint. The remaining subscales clustered in the lower-moderate range (medians 2.40–3.00 in students and 2.40–2.88 in staff), so that neither marked emotional eating nor marked food avoidance predominated. Among students, the widest gender gaps were visible for SR (median 3.00 in women compared with 2.50 in men), EOE (2.80 compared with 2.20), H and SE, whereas the EF, FF and FR distributions overlapped almost completely. Staff medians for EOE (2.80) exceeded those of students (2.40), whereas EF medians were lower. Complete descriptive statistics, with the number of valid responses, are given in Table S1.

### 3.5. Gender Differences in Appetitive Traits

The unequal gender composition of the two cohorts requires gender differences to be examined within cohorts. Mann–Whitney *U* tests were therefore conducted separately for students and staff across the eight subscales, with a Bonferroni-corrected threshold of *p* < 0.00625 (Table 5).

Among students, gender differences reached the corrected threshold for five of the eight subscales, women scoring higher in every case: SR (*r* = 0.278), H (*r* = 0.209), EUE (*r* = 0.204), EOE (*r* = 0.189) and SE (*r* = 0.164). All effects were small, so gender accounts for a modest but consistent share of variance. EF, FF and FR showed negligible differences (*r* = 0.042, 0.062 and 0.017), indicating that food enjoyment, fussiness and responsiveness to external food cues were similarly distributed between male and female students.

Among staff, no subscale reached the corrected threshold, most likely reflecting the smaller cohort (*n* ≤ 41 men and 72 women per subscale). The direction was nevertheless consistent with students for EOE (*r* = 0.091) and EUE (*r* = 0.102); the only nominally significant staff difference ran the other way, men scoring higher on FF (*p* = 0.020, *r* = 0.219).

### 3.6. Cohort Differences and Emotional-Eating Profiles

Students and academic staff differ in age, daily routine and occupational demands, so cohort was examined as a second source of variation in appetitive traits. Mann–Whitney *U* tests compared the two cohorts across the eight subscales, with the same Bonferroni-corrected threshold (Table 6).

Students scored higher on EF (3.76 ± 0.82 compared with 3.29 ± 0.77; *p* < 0.001, *r* = 0.216), whereas staff reported higher EOE (2.79 ± 0.88 compared with 2.52 ± 0.96; *p* = 0.003, *r* = 0.111). The higher SR (2.83 ± 0.59 compared with 2.65 ± 0.69; *p* = 0.009) and lower FR (*p* = 0.034) of staff did not survive the correction for eight comparisons, and EUE, FF, SE and H did not differ between cohorts. Both cohort effects were small and confined to hedonic engagement and emotional over-eating, leaving external responsiveness, hunger and the food-avoidance traits unaffected.

**Figure 3:**
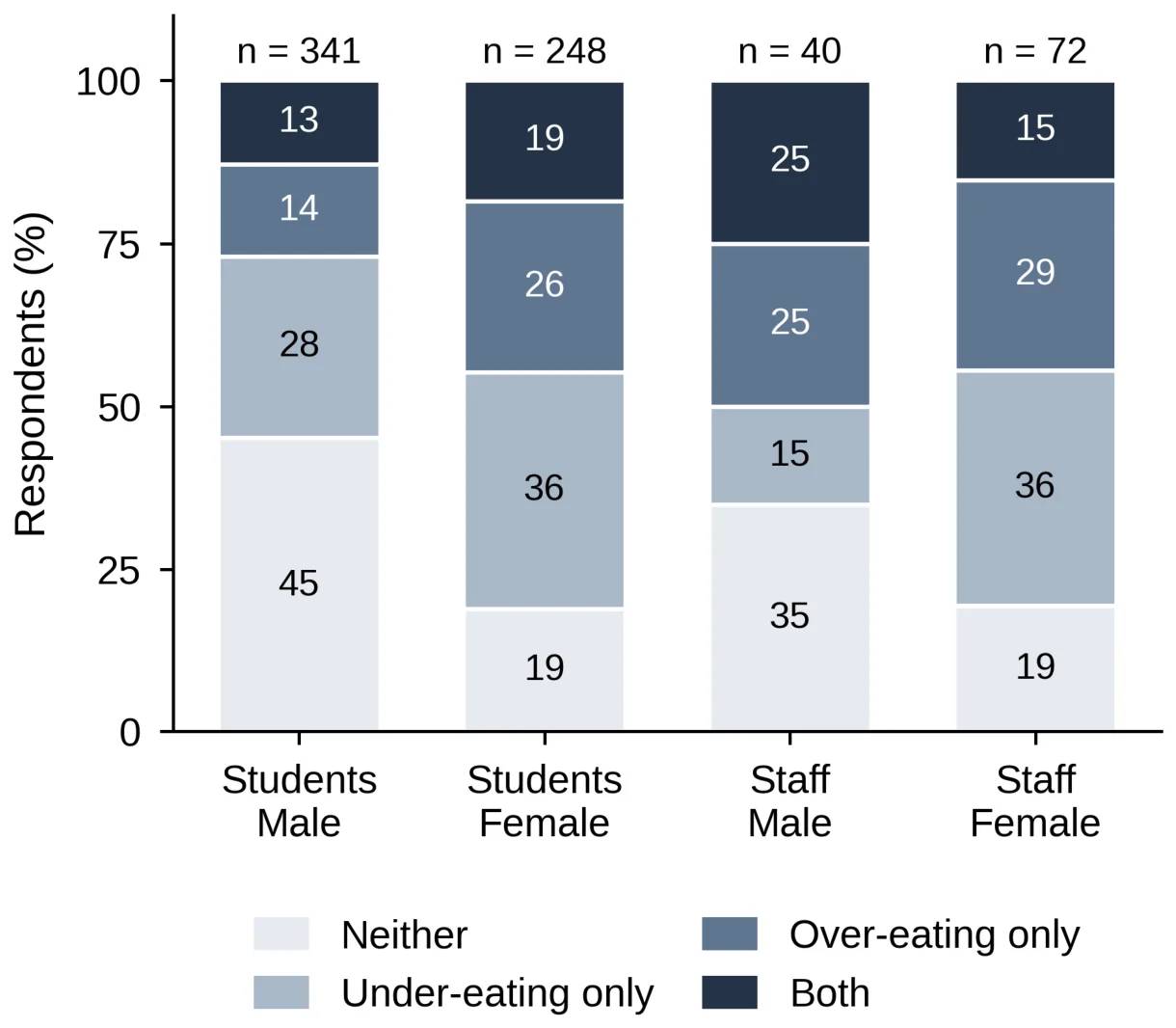
Emotional-eating profiles by cohort and gender. Profiles defined from the Emotional Over-Eating (EOE) and Emotional Under-Eating (EUE) subscales of the Adult Eating Behaviour Questionnaire: over-eating only (EOE ≥ 3, EUE < 3), under-eating only (EOE < 3, EUE ≥ 3), both (EOE ≥ 3, EUE ≥ 3) or neither (both < 3). Percentages within bars (segments below 7% unlabelled); shading from light to dark: neither, under-eating only, over-eating only, and both; *n* above bars.

EOE and EUE describe opposite responses to the same emotional states; the two subscales were therefore combined into a four-category profile using the agreement side of the scale (score ≥ 3). Emotional over-eating without under-eating characterised 19.2% of students and 27.7% of staff; under-eating without over-eating, 31.4% and 28.6%; both responses, 15.3% and 18.8%; and neither, 34.1% and 25.0% (Figure 3).

The profile distribution differed by gender (χ^2^(3) = 51.64, *p* < 0.001, Cramér’s V = 0.271) but not by cohort (χ^2^(3) = 6.66, *p* = 0.083, Cramér’s V = 0.098). The gender contrast arose mainly from the “neither” category, reported by 45.2% of male students compared with 19.0% of female students and by 35.0% of male staff compared with 19.4% of female staff. Among students, the excess among women was spread across all three reactive categories, whereas among staff, it was concentrated in under-eating only (36.1% compared with 15.0%); in neither cohort, therefore, did the higher mean EOE of women reflect a shift towards over-eating specifically. The non-significant cohort difference, by contrast, was confined to the over-eating categories (over-eating only 27.7% of staff compared with 19.2% of students), with under-eating only slightly less frequent among staff.

### 3.7. COVID-19 Infection Status and Appetitive Traits

Confirmed infection was reported by nearly half of staff and by a third of students, so infection history was examined as a further source of variation in appetitive traits. Kruskal–Wallis tests compared the eight subscale scores across three status groups (confirmed infection, no confirmed infection and unknown) within each cohort, with the Bonferroni-corrected threshold (Table 7).

Among students, the only effect surviving correction concerned H (*H* = 13.43, *p* = 0.001, *η*^2^ = 0.020): students with confirmed infection reported higher hunger (3.05 ± 0.79) than those without (2.77 ± 0.73), a small effect. No other subscale differed by infection status in either cohort; in staff, H differed nominally (*H* = 6.22, *p* = 0.045, *η*^2^ = 0.039) but not at the corrected threshold, with the highest scores in the small “unknown” group (*n* = 14). Infection history was therefore not broadly associated with appetitive traits. The corresponding comparison across physical-activity categories is provided in Table S4.

### 3.8. Correlates of Appetitive Traits: Physical Activity, Perceived Health and Mental Health

Bivariate associations between the appetitive traits and the lifestyle and health variables were examined next. Spearman correlations were computed, separately for students and staff, between the eight subscale scores and physical activity in both periods and its change, current self-rated health, perceived health change, the mental health score and the SF-36-derived limitation, social-interference, pain and health-perception indices (Table 8).

Physical activity during the pandemic was inversely associated with EOE in both cohorts (students *ρ* = −0.162, staff *ρ* = −0.230; Figure S1) and, more weakly, with H and SR in students. Pre-pandemic activity was unrelated to EOE (students *ρ* = −0.046, staff −0.154), and the individual change in activity showed only a small inverse association with EOE in students (*ρ* = −0.091) that was not significant in staff (−0.133); in staff, the change in activity was instead inversely related to SE (*ρ* = −0.317), EUE and SR.

The mental health score was the most consistent correlate, inversely related to H (students *ρ* = −0.306, staff −0.358), EOE (−0.222 and −0.189), EUE, FR and SR in both cohorts, and emotional role limitations showed the mirror pattern (EOE 0.226 and 0.218; H 0.191 and 0.323). Physical limitations, social interference and pain impact were positively associated with EOE and H in students and, for the last two, in staff as well. Current self-rated health correlated with EOE only weakly in students (−0.115) and more strongly in staff (−0.276), whereas the perceived change in health since the pre-pandemic period was unrelated to EOE in either cohort (−0.059 and 0.085). EF was largely independent of the health variables, apart from small positive associations with self-rated health and the health-perception score in students. Correlations were generally of small magnitude, the largest being observed in staff between FR and pain impact (*ρ* = 0.412) and between H and the mental health score (*ρ* = −0.358).

After Benjamini–Hochberg adjustment within each cohort, 34 of the 38 nominally significant correlations in students remained significant at *q* < 0.05, the exceptions being the associations of SR with pandemic activity, of EOE with the change in activity and of FR with physical limitations and current self-rated health. In staff, 11 of 27 remained: the associations of H with the mental health score, emotional role limitations, social interference, physical limitations and pain impact, of FR with the mental health score, emotional role limitations and pain impact, of EOE with current self-rated health, of FF with social interference and of SE with the change in activity. The other staff correlations of EOE, including those with pandemic physical activity, the mental health score and emotional role limitations (*q* = 0.09–0.15), therefore do not withstand correction for multiple testing and are treated as exploratory; adjusted values for all correlations are given in Table S8.

### 3.9. AEBQ Subscale Intercorrelations

**Figure 4:**
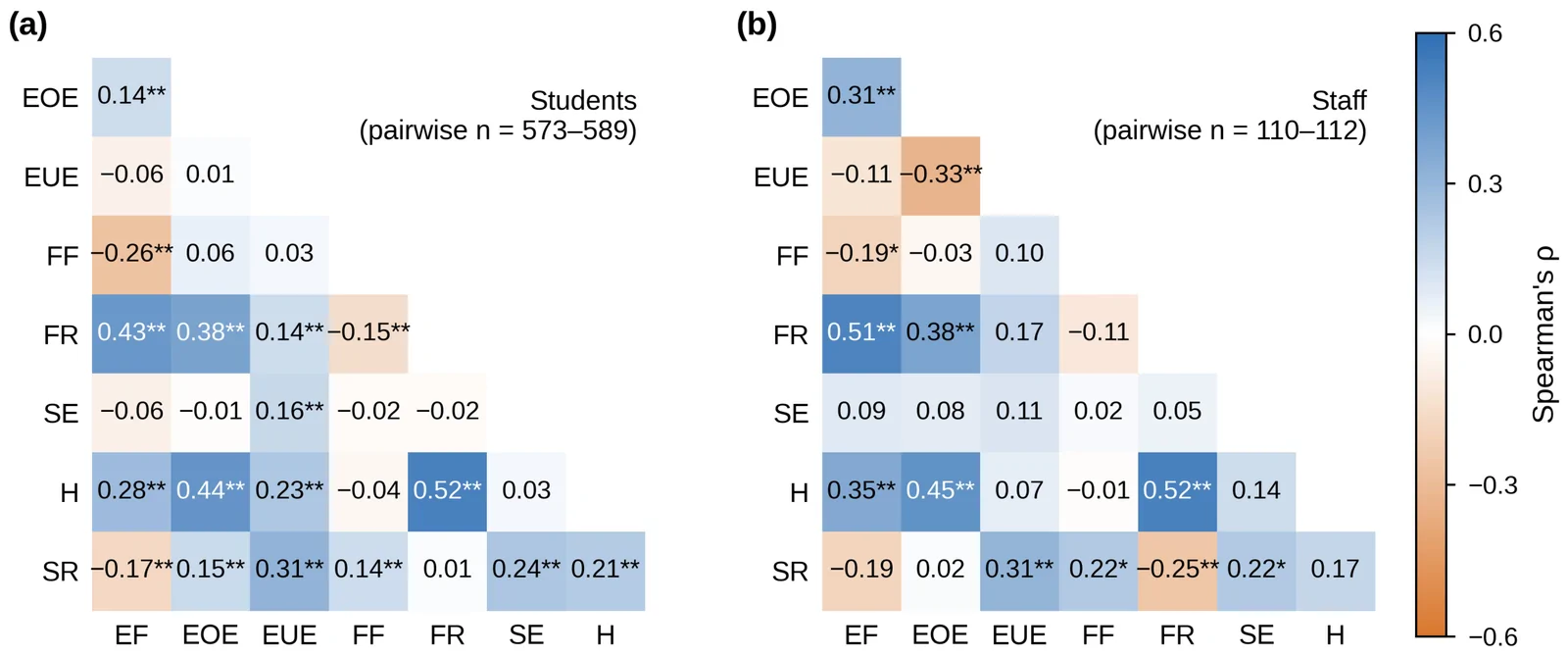
Spearman correlation matrices of AEBQ subscale scores in students and staff. (**a**) Students; (**b**) staff. Lower triangle of the matrix; cell shading encodes Spearman’s *ρ* (blue: positive; orange: negative; white: zero), values printed in each cell; ** *p* < 0.01, * *p* < 0.05, unadjusted for multiple testing (exploratory comparisons; Benjamini–Hochberg *q* values in Table S8); pairwise *n* range in each panel. EF: Enjoyment of Food; EOE: Emotional Over-Eating; EUE: Emotional Under-Eating; FF: Food Fussiness; FR: Food Responsiveness; SE: Slowness in Eating; H: Hunger; SR: Satiety Responsiveness; AEBQ: Adult Eating Behaviour Questionnaire.

The pattern of associations among the eight traits indicates whether the food-approach and food-avoidance dimensions behave as expected in this sample. Spearman correlation matrices were therefore computed separately for students and staff (Figure 4).

A food-approach cluster was evident in both cohorts: the strongest associations linked FR with H (students *ρ* = 0.52; staff *ρ* = 0.52), EOE with H (0.44; 0.45) and EF with FR (0.43; 0.51). EF was negatively related to FF (−0.26; −0.19) and, in students, to SR (−0.17), confirming the expected opposition between hedonic engagement and food-avoidance traits. SE behaved as a food-avoidance trait, correlating positively with SR (0.24; 0.22) and, in students, with EUE (0.16), while the remaining associations among the food-avoidance traits were weak. The clearest divergence between cohorts concerned the EOE–EUE relationship: essentially uncorrelated among students (*ρ* = 0.01) but moderately negative in staff (*ρ* = −0.33), so that in the older working cohort the two emotional responses tended to exclude each other. All correlations described above remained significant after FDR adjustment except the staff EF–FF correlation (*q* = 0.111). Correlations in the combined sample are provided in Table S5 and FDR-adjusted values in Table S8.

### 3.10. Independent Correlates of Emotional Over-Eating

The bivariate associations above do not separate the contribution of gender, cohort, pandemic-related change and psychological state, which are themselves interrelated. Two regression approaches were therefore used: ordinary least squares regression with HC3 standard errors for the EOE score (primary analysis) and binary logistic regression for high EOE (score ≥ 3.5; secondary analysis). The primary model (Model 1) contains only variables external to the AEBQ; Model 2 adds EF and EUE as a sensitivity analysis (Table 9).

The primary model was significant but explained a modest share of variance (*R*^2^ = 0.127, *F*(8, 581) = 10.17, *p* < 0.001; *n* = 590). Four predictors were independently associated with EOE: female gender (*B* = 0.243, 95% CI: 0.085 to 0.400, *p* = 0.003), a lower mental health score (*B* = −0.118, *p* = 0.012), more emotional role limitations (*B* = 0.129, *p* < 0.001) and lower current physical activity (*B* = −0.083 per SD of log MET-min/week, *p* = 0.037); the staff cohort effect fell just short of significance (*B* = 0.198, *p* = 0.058). Neither the pandemic-related changes, namely the reported individual decline in physical activity (*p* = 0.298) and the perceived change in health (*p* = 0.386), nor confirmed infection (*p* = 0.476) contributed once gender, cohort, psychological state and current activity were taken into account. Equivalence tests supported the absence of a material effect for the reported decline in physical activity (90% CI: −0.095 to 0.021 per SD; TOST *p* = 0.036) and for the perceived change in health (90% CI: −0.097 to 0.030 per scale unit; *p* = 0.042), both intervals lying within ±0.10 EOE points, whereas the interval for confirmed infection (90% CI: −0.076 to 0.191) exceeded the upper bound (*p* = 0.300), so a small positive association with infection cannot be excluded. With the alternative margins, equivalence for the two lifestyle changes also held at ±0.15 and ±0.20 (TOST *p* ≤ 0.001) but not at ±0.05 (*p* = 0.353 and 0.334), and for confirmed infection only at ±0.20 (*p* = 0.040). Adding EF and EUE (Model 2) raised the explained variance to 0.195 and enlarged the gender and cohort coefficients (*B* = 0.301 and 0.306), with EF positively (*B* = 0.240) and EUE negatively (*B* = −0.180) related to EOE; the pattern of the external predictors was unchanged. Variance inflation factors were below 1.5 in both models.

The logistic models examined the same predictors for the odds of a score in the upper range of the EOE scale (≥3.5), reached by 102 of 590 respondents with complete data (Table 10).

In the primary logistic model, women had more than twice the odds of high EOE (OR = 2.18, 95% CI: 1.35–3.52, *p* = 0.002); each additional emotional role limitation was associated with about 40% higher odds (OR = 1.39, *p* = 0.004) and a higher mental health score with lower odds (OR = 0.76, *p* = 0.024). Current physical activity did not reach significance in this model (OR = 0.82 per SD of log MET-min/week, *p* = 0.072), nor did the staff cohort (OR = 1.42, *p* = 0.255), infection status, the change in physical activity or the perceived change in health. In Model 2, the gender coefficient increased (OR = 3.35), and the staff effect remained non-significant (OR = 1.81, *p* = 0.081), while EUE was strongly inversely (OR = 0.34) and EF positively (OR = 1.47) associated with high EOE; Nagelkerke *R*^2^ rose from 0.150 to 0.307.

With the lower threshold for high EOE (score ≥ 3.0; 214 events), the four predictors of the linear model were all associated with high EOE: female gender (OR = 1.56, *p* = 0.018), emotional role limitations (OR = 1.33, *p* < 0.001), the mental health score (OR = 0.81, *p* = 0.034) and current physical activity (OR = 0.80, *p* = 0.016). With the stricter threshold (score ≥ 4.0; 57 events), only the mental health score remained significant (OR = 0.63, 95% CI: 0.46–0.86, *p* = 0.004); the estimates for female gender (OR = 1.39, *p* = 0.286) and emotional role limitations (OR = 1.24, *p* = 0.136) kept their direction but lost significance, as expected from the smaller number of events.

Rescoring the emotional role limitations count from complete item sets only (*n* = 547) or prorated to the number of answered items changed the Model 1 coefficients by at most 0.02 and left the pattern of significant and non-significant predictors unchanged. In the age-overlapping subgroup (*n* = 89 students and 14 staff with EOE scores), the cohort difference in EOE was not significant (2.45 ± 0.90 compared with 2.70 ± 1.02; *U* = 515.0, *p* = 0.299, *r* = 0.102), and the cohort coefficient of Model 1 refitted in this subgroup was 0.24 (95% CI: −0.37 to 0.84; *n* = 87), close to the full-sample estimate but with a wide interval. Within cohorts, EOE decreased slightly across age groups in students (*ρ* = −0.095, *p* = 0.021) and showed no age trend in staff (*ρ* = 0.025, *p* = 0.797), whereas EF and H decreased with age in staff (*ρ* = −0.302, *p* = 0.001 and *ρ* = −0.221, *p* = 0.019). Full results of the sensitivity and age analyses are given in Tables S9 and S10.

Taken together, the two models identify female gender and current psychological state (a lower mental health score and emotional role limitations) as the independent correlates of emotional over-eating that were consistent across the linear and logistic models; current physical activity showed a small inverse association in the linear model and at the ≥3.0 threshold but not at ≥3.5, and the cohort effect was borderline in the linear model only. The pandemic-related variables available at the individual level, namely the reported decline in physical activity, the perceived deterioration of health and confirmed infection, added no detectable contribution once these factors were accounted for, and for the two lifestyle changes, the equivalence tests excluded effects larger than ±0.10 scale points. The corresponding models for SR and H are given in Table S6.

## 4. Discussion

The present study examined appetitive traits, pandemic-related change in physical activity and perceived health, and their interrelations in two institutionally paired but demographically distinct university cohorts, using the AEBQ and a multivariable framework in which pandemic-related change was measured at the individual level. Three findings organise the discussion: respondents reported a marked decline in physical activity since the pre-pandemic period and, especially among staff, a deterioration of perceived health, whereas the appetitive-trait profile recorded during the pandemic was in the lower-moderate range; emotional over-eating was associated with gender and psychological state more than with the reported pandemic-related changes; and the Romanian AEBQ reproduced the expected eight-factor structure, with internally consistent emotional-eating subscales.

### 4.1. Appetitive-Trait Profiles, Pandemic-Related Change and Cohort Differences

Across both cohorts, EF was the only trait above the scale midpoint; emotional eating and food-avoidance subscales were in the lower-moderate range, and only about one in five students and one in four staff reported emotional over-eating without under-eating. Neither emotional eating nor food avoidance was therefore predominant in the profile recorded during the pandemic, and the retrospectively reported halving of median physical activity and the perceived deterioration of health (one in five students and more than one in three staff) coexisted with this profile. European studies reported pronounced increases in emotional eating during lockdown periods [41,42], and a meta-analysis found a small overall increase [9]; whether a similar change occurred in this sample cannot be established from a single measurement, and the lower-moderate level observed here may reflect cultural differences in coping, the timing of data collection (a hybrid teaching period rather than a lockdown) or selection towards more resilient respondents in a voluntary survey. This pattern is also consistent with the multivariable results, in which neither the reported individual decline in physical activity nor the perceived deterioration of health was related to emotional over-eating once gender and psychological state were accounted for, and equivalence tests bounded their coefficients within ±0.10 scale points: the reported lifestyle changes and emotional over-eating appear to have been unrelated at the individual level.

The null result for the pandemic-related changes could in principle reflect recall attenuation, because the pre-pandemic values were reported retrospectively. Two features of the data argue against this explanation as the whole story. First, current physical activity, reported contemporaneously, was itself associated with EOE, so the change score was not simply too noisy to carry any signal. Second, the perceived deterioration of health was measured by a direct comparative item that does not depend on recalling a numerical pre-pandemic value, yet it was equally unrelated to EOE. The equivalence tests further bounded both coefficients within a tenth of a scale point, so only an association that was already small before any attenuation could have been concealed.

The higher EF among students (*r* = 0.216) is consistent with age-related declines in food enjoyment [22] and likely reflects the hedonic food engagement characteristic of younger adults in social eating contexts [43]. The higher EOE among staff (*r* = 0.111) is in the direction of the increase in stress-related eating reported among working-age adults under stay-at-home conditions [44]. This difference was, however, attenuated to a borderline effect after adjustment for gender and psychological state (*p* = 0.058) and did not translate into higher odds of high EOE. The higher SR of staff did not survive correction for multiple comparisons, although its direction is consistent with age-related increases in satiety sensitivity [45,46]. In the age-overlapping subgroup, the cohort difference in EOE was not significant, and the adjusted coefficient was similar to the full-sample estimate but imprecise; cohort should therefore be regarded as a secondary correlate at most, not separable from age.

The combined emotional-eating profile qualifies what mean scores conceal. Under-eating in response to emotion was at least as common as over-eating in every group except male staff. The higher EOE of women reflected a larger share of respondents reporting any emotional modulation of intake rather than a shift towards over-eating specifically. The small cohort difference, by contrast, was confined to the over-eating categories. The cohorts also differed in how the two responses related to each other: independent in students but inversely related in staff. In the older working cohort, emotional eating thus tended to consolidate into a directional style, either over- or under-eating, whereas young adults more often reported both. Whether this reflects age, occupational stress or the chronicity of stress exposure remains open, but it suggests that, if confirmed prospectively, interventions for working adults may need to distinguish the direction of the emotional response.

### 4.2. Gender, Infection Status and Physical Activity

Gender was the most consistent correlate of appetitive traits. Female students scored higher than males on EOE, EUE, H, SR and SE (*r* = 0.164–0.278), replicating patterns documented across cultural contexts [15,16,47]. These patterns have been confirmed meta-analytically for the pandemic period, with a moderate pooled effect and heightened vulnerability among young adults [19], and the present data extend them to a young Eastern European population. Effect sizes were uniformly small, and the profile analysis locates the difference mainly in the proportion of women reporting any emotional modulation of intake (81% compared with 55% of male students) rather than in over-eating specifically. The association with gender was robust for the EOE score and for high EOE at the ≥3.0 and ≥3.5 thresholds, but not at ≥4.0, where only the mental health score remained associated with high EOE; with 57 events, that model has limited power, so a gender difference in the most pronounced over-eating is unconfirmed rather than excluded. The absence of gender differences in EF and FR is consistent with the original AEBQ validation [22], suggesting that hedonic food engagement and external cue sensitivity are similarly distributed between genders.

Confirmed COVID-19 infection was associated with higher Hunger scores among students (*η*^2^ = 0.020), the only infection-related finding across the subscales, and one that did not extend to EOE in the multivariable models. Post-COVID metabolic and appetite dysregulation has been documented clinically [48], including altered taste and smell and gastrointestinal sequelae that could plausibly alter appetite perception. The effect was small, and the nominal staff result (*p* = 0.045, not significant after correction) arose from the small group of uncertain infection status rather than from confirmed infection, so it does not corroborate the student finding. Prospective replication with biological markers of appetite regulation is warranted.

Physical activity during the pandemic was inversely associated with EOE in students (*ρ* = −0.16, significant after FDR adjustment) and, as an exploratory finding, in staff (*ρ* = −0.23, not significant after adjustment), and remained independently, though weakly, associated with the EOE score in the linear model and at the ≥3.0 threshold, but not at ≥3.5, in line with evidence that regular physical activity improves appetite regulation and the control of energy intake [17,18]. In a recent large student sample, the inverse association between exercise and emotional eating was moderate and partly mediated by coping style and emotion regulation [49]. The reported individual decline in activity between the pre-pandemic and pandemic periods, however, was unrelated to EOE once current activity was accounted for, which suggests that the association reflects a stable relationship between an active lifestyle and emotional eating rather than an acute consequence of the reported pandemic-related loss of activity.

### 4.3. Psychological State and Emotional Over-Eating

Psychological state, captured by the mental health score and by emotional role limitations, was the strongest correlate of emotional over-eating apart from gender, in both the linear and the logistic models, the two indicators being associated with it independently of each other. This pattern is consistent with the role of psychological distress in emotional over-eating posited by the five-way model of emotion and eating [16] and the stress–reward account of overeating [50], and with pandemic-era evidence from Italian lockdown studies [42]. The parallel association between mental health and H (*ρ* = −0.31 in students and −0.36 in staff) suggests that distress may relate to appetite through two pathways, heightened emotional reactivity to food and increased perceived hunger, rather than through a single mechanism.

The gender asymmetry in psychological indicators documented in this sample (women reporting lower mental health scores and more emotional role limitations in both cohorts) provides a plausible partial explanation for the gender difference in EOE. Women’s greater vulnerability to stress-induced eating is well established in the literature [16,47], and the present findings are consistent with psychological distress, more than perceived physical health, accounting for part of this difference: current self-rated health was unrelated to EOE when added to the primary model (Table S6), and the perceived deterioration of health since the pre-pandemic period, although far more frequent among staff and among women, was not associated with emotional over-eating.

Contextual stressors specific to academic life may also have contributed to the psychological state captured here. Examination periods are a documented source of academic stress and have been associated with changes in students’ eating behaviour, including irregular meal timing and increased consumption of sweet foods [51], and academic staff face recurrent periods of elevated occupational demand linked to administrative reporting, accreditation, performance evaluation and teaching load. The survey took place in October–November 2021, within the teaching semester; to the extent that such stressors act through the same psychological pathway, part of the association between psychological state and emotional over-eating may reflect academic and occupational stress rather than the pandemic itself, a possibility that repeated measurement across the academic calendar could resolve.

These findings suggest potential implications for university health and nutrition services, which require prospective confirmation. National nutrition policy in Moldova has so far addressed food security and dietary quality rather than the behavioural and psychological determinants of eating [52]. Because emotional over-eating was associated with psychological state more than with the reported loss of physical activity or perceived ill-health, nutrition education alone may not reach the students and staff most affected; screening for emotional eating could be paired with accessible mental-health support, and the promotion of physical activity, which by the respondents’ own account fell sharply during the pandemic, could be framed as relevant to mood and eating regulation as well as to energy balance. Women and respondents reporting emotional role limitations were the groups with the highest emotional over-eating scores in these data.

### 4.4. Reliability and Structure of the AEBQ in a Romanian-Speaking Sample

Prior cross-cultural validations of the AEBQ have reported adequate to good reliability across diverse linguistic contexts [22,23,25,26,27], yet Romanian-speaking populations had not been examined before the present study. The Romanian versions of the Dutch Eating Behavior Questionnaire and of the Emotional Eater Questionnaire have, however, reported good reliability for their emotional-eating scales [28,29], in line with the high reliability of EOE and EUE observed here. The eight-factor structure was supported with an estimator appropriate for ordinal items, and EOE, EUE and SE showed the highest *α* values (0.905, 0.871 and 0.813 in students), matching or exceeding the original validation and supporting their use as outcome measures. EF and H met the acceptability threshold, while FF, FR and SR fell below 0.70, a pattern previously observed in Mexican [25] and Chinese [26] samples, suggesting that these subscales are more susceptible to linguistic and cultural adaptation effects than the emotional eating dimensions. More recent adaptations point in the same direction: Hunger and Satiety Responsiveness were the weakest or unstable subscales in Turkish, Arabic and United States samples [53,54,55], whereas a Chinese university sample retained all eight factors with *α* > 0.70 [56].

Two features of the Romanian version deserve explicit mention because they affect how the instrument should be used. First, SE contains a single reverse-keyed item (“I often finish my meals quickly”); in a preliminary analysis of these data, a second SE item had been reversed in error, producing a negative *α* that could easily have been misread as a cultural artefact, whereas the correctly scored subscale is among the most internally consistent. Item-level checks of keying direction are therefore indispensable before reliability coefficients are interpreted cross-culturally. Second, one EUE item was administered with the same Romanian wording as another item of the subscale, so EUE was scored on four unique items; the missing item (“I eat less when I’m irritable”) should be restored in future use of the Romanian version.

The below-threshold reliability of FF, FR and SR, together with the weak loadings of two FF items (“I often decide that I don’t like a food, before tasting it” and “I refuse new foods at first”) and one H item, means that findings involving these subscales should be regarded as exploratory and point to the adaptation work still needed. The weak H item illustrates the problem: the original “If I miss a meal I get light-headed” was rendered in Romanian as a headache when meals are missed, a different symptom, which may explain its low loading (0.43). The change-in-fit criteria for metric and scalar invariance were met across gender and, with items treated as continuous, across cohort, which supports the between-group comparisons reported here within the limits of a configural fit below the conventional cut-offs. Cognitive interviewing and rewording of the weakest items, followed by item-level invariance testing in a larger staff sample, are the next steps before the Romanian version can be regarded as validated.

### 4.5. Limitations

Several limitations should be considered. The cross-sectional design precludes causal inference for all observed associations; in particular, the direction of the relationship between psychological state and emotional over-eating cannot be established from these data. Convenience sampling from a single institution, without a participation rate (Section 2.1), limits generalisability beyond TUM: the percentages reported here describe the respondents and are not estimates for Moldovan university populations. Voluntary participation may also have selected more health-conscious or resilient respondents, attenuating the prevalence of maladaptive eating. The staff cohort was small (*n* = 121), which reduced power for within-staff comparisons and made the staff-only factor model unstable; moreover, cohort and age are confounded by design, and the age-overlapping subgroup contains only 15 staff, so cohort differences cannot be separated from age differences or attributed to occupational context. No a priori sample-size calculation was performed; with 590 complete cases, the linear model had a power of 0.93 to detect a small effect (Cohen’s *f*^2^ = 0.02) of a single predictor at *α* = 0.05, with a minimum detectable effect of *f*^2^ ≈ 0.014 at 80% power, whereas the logistic models had 26.8, 12.8 and 7.1 events per predictor at the ≥3.0, ≥3.5 and ≥4.0 thresholds, so the ≥4.0 model is under-powered and its null results for gender and emotional role limitations are inconclusive. Missing responses were handled by pairwise deletion; the effective *n* is reported for every estimate, and the primary regression models rest on 590 respondents with complete data. Unanswered yes/no items within an answered block were counted as not endorsed, a rule that may understate role limitations. The Romanian AEBQ was translated directly by the research team without back-translation or pilot testing, which is the most likely origin of the duplicated EUE item and of the altered H item and a plausible contributor to the lower reliability of FF, FR and SR; measurement invariance across gender met the change-in-fit criteria but not the χ^2^ difference tests, the configural model fitted below the conventional cut-offs, and invariance across cohort could be tested only with items treated as continuous because of the small staff group, so partial non-equivalence of item parameters between genders and between cohorts cannot be excluded and may have contributed to the observed group differences. The health-perception score had only modest internal consistency (*α* = 0.60–0.66) and was used in the correlational analyses only. The mental health score and the emotional role limitations count were derived from SF-36 items but do not reproduce the validated SF-36 scales or their scoring. They are therefore study-specific indicators of psychological state rather than standardised measures, although the mental health score was internally consistent (*α* = 0.90).

The exclusive reliance on self-report instruments introduces measurement bias, as respondents may underreport socially undesirable behaviours such as emotional over-eating; because all variables were obtained from the same questionnaire at one sitting, common-method variance may also have inflated the associations between psychological state and emotional over-eating. Academic and occupational stressors specific to the survey period were not measured. The absence of anthropometric data (body mass index, body weight) precludes examination of the relationship between eating behaviour traits and weight status, which represents an important gap given the established links between emotional eating and obesity risk [21,57]. The AEBQ was administered once, during the pandemic, without a pre-pandemic measurement or a contemporaneous comparison group, so the study describes appetitive traits under pandemic conditions and cannot determine whether they changed. The pre-pandemic values of physical activity and health were reported retrospectively, so the within-person changes are subject to recall bias and to the possibility that pandemic-period mood coloured the recollection of the past; the change estimates also rest on the respondents who answered for both periods (84% of the sample). Physical activity was self-reported with duration in categories converted to fixed values, which adds imprecision to the MET-min/week estimates, and scored according to the GPAQ protocol, so the prevalence of inactivity is not directly comparable with surveys that also score light activity. The data were collected in autumn 2021. Although the pandemic context is explicitly modelled here, the trait–correlate associations should be replicated under non-pandemic conditions, the more so as student mental health had not returned to pre-pandemic levels by 2022 in longitudinal cohorts [14].

## 5. Conclusions

Within the ±0.10 equivalence margin, the individual-level pandemic changes that respondents reported in physical activity and perceived health carried no independent association with emotional over-eating once gender, cohort and psychological state were accounted for. Emotional over-eating in this sample of Moldovan university students and staff is therefore better understood as a correlate of gender and psychological state than of the reported pandemic-related lifestyle disruption; current physical activity shows only a small inverse association, and cohort at most a secondary one.

On this preliminary evidence, the Romanian AEBQ can be used for its emotional over-eating, emotional under-eating and slowness in eating subscales, whereas food fussiness, food responsiveness and satiety responsiveness require further adaptation, and one emotional under-eating item must be restored and one hunger item reworded before the version is used again.

For university health and nutrition services, these findings suggest, pending prospective confirmation, that emotional over-eating may be better addressed together with mental health than through nutrition education alone, with particular attention to women and to respondents reporting emotional role limitations, and that the promotion of physical activity could belong in the same programme. Longitudinal designs with anthropometric and biological markers, and item-level invariance testing of the Romanian AEBQ in larger staff samples, are the next steps.

## Figures and Tables

**Table 1:** Sample characteristics by cohort and gender.

Variable	Students Male (*n* = 362)	Students Female (*n* = 261)	Students Total (*n* = 623)	Staff Male (*n* = 42)	Staff Female (*n* = 79)	Staff Total (*n* = 121)
Age group, *n* (%)						
18–20	176 (48.6)	118 (45.2)	294 (47.2)	—	—	—
21–23	128 (35.4)	99 (37.9)	227 (36.4)	—	—	—
24–26	23 (6.4)	19 (7.3)	42 (6.7)	—	—	—
>26	33 (9.1)	25 (9.6)	58 (9.3)	—	—	—
<25	—	—	—	4 (9.5)	5 (6.3)	9 (7.4)
26–30	—	—	—	3 (7.1)	3 (3.8)	6 (5.0)
31–40	—	—	—	10 (23.8)	20 (25.3)	30 (24.8)
41–50	—	—	—	10 (23.8)	30 (38.0)	40 (33.1)
51–60	—	—	—	5 (11.9)	15 (19.0)	20 (16.5)
61–70	—	—	—	7 (16.7)	4 (5.1)	11 (9.1)
>70	—	—	—	3 (7.1)	2 (2.5)	5 (4.1)
Not reported	2 (0.6)	0 (0.0)	2 (0.3)	0 (0.0)	0 (0.0)	0 (0.0)
COVID-19 infection, *n* (%)						
Confirmed	100 (27.6)	92 (35.2)	192 (30.8)	18 (42.9)	39 (49.4)	57 (47.1)
Not confirmed	167 (46.1)	111 (42.5)	278 (44.6)	19 (45.2)	30 (38.0)	49 (40.5)
Unknown	94 (26.0)	58 (22.2)	152 (24.4)	5 (11.9)	10 (12.7)	15 (12.4)
Not reported	1 (0.3)	0 (0.0)	1 (0.2)	0 (0.0)	0 (0.0)	0 (0.0)
Physical activity, pandemic period, *n* (%) ^1^						
Inactive	84 (25.5)	91 (37.4)	175 (30.6)	12 (33.3)	26 (36.1)	38 (35.2)
Minimally active	123 (37.4)	104 (42.8)	227 (39.7)	13 (36.1)	29 (40.3)	42 (38.9)
Health-enhancing	122 (37.1)	48 (19.8)	170 (29.7)	11 (30.6)	17 (23.6)	28 (25.9)
MET-min/week, median [IQR] ^1^	1800 [480–4320]	960 [240–2400]	1440 [360–3840]	1860 [360–4860]	1020 [210–2520]	1200 [240–3180]
Self-perceived health						
Current health, 1–5, mean ± SD ^2^	3.24 ± 1.13	2.84 ± 1.02	3.07 ± 1.10	2.85 ± 0.94	2.84 ± 0.94	2.84 ± 0.94
Health compared with pre-pandemic, worse/same/better, %	19/50/31	26/42/33	22/47/32	31/50/19	41/35/24	37/40/22
Mental health score, 1–6, mean ± SD ^3^	3.96 ± 1.01	3.51 ± 1.09	3.77 ± 1.07	4.25 ± 1.00	3.89 ± 1.07	4.01 ± 1.05
Emotional role limitations, 0–3, mean ± SD ^4^	1.42 ± 1.20	1.75 ± 1.16	1.56 ± 1.19	0.93 ± 1.01	1.27 ± 1.12	1.15 ± 1.09

Note. IQR: interquartile range; MET: metabolic equivalent of task; SD: standard deviation. Percentages refer to the column *n* unless stated otherwise. Age categories differ between the two questionnaires. Physical activity: moderate-to-vigorous MET-min/week (GPAQ scoring); categories according to GPAQ thresholds: inactive <600, minimally active 600–3000, health-enhancing >3000 MET-min/week. ^1^ Percentages of valid responses after removal of implausible reports (>16 h/day): students *n* = 572, staff *n* = 108. ^2^ 1 = poor to 5 = excellent; students *n* = 548, staff *n* = 114. ^3^ Composite of eight SF-36-derived items, higher = better mental health; students *n* = 618, staff *n* = 121. ^4^ Number of endorsed role limitations due to emotional problems; students *n* = 617, staff *n* = 119. Health compared with pre-pandemic: the health-comparison item, all respondents. GPAQ: Global Physical Activity Questionnaire; SF-36: 36-item Short Form Health Survey.

**Table 2:** Physical activity before and during the pandemic in respondents with paired data.

Cohort	*n*	Pre-Pandemic MET-min/Week, Median [IQR]	Pandemic MET-min/Week, Median [IQR]	Decreased/Unchanged/Increased, %	Health-Enhancing, Pre-Pandemic, %	Health-Enhancing, Pandemic, %	*p * ^1^	*r*
Students	524	3120 [1290–6480]	1440 [360–3360]	53.2/33.4/13.4	51.5%	28.4%	<0.001	0.502
Staff	103	2160 [780–5880]	1200 [240–3000]	47.6/40.8/11.7	39.8%	25.2%	<0.001	0.502
Combined	627	2880 [1200–6240]	1320 [360–3360]	52.3/34.6/13.1	49.6%	27.9%	<0.001	0.500

Note. IQR: interquartile range; MET: metabolic equivalent of task. Moderate-to-vigorous activity (GPAQ scoring); health-enhancing: >3000 MET-min/week. ^1^ Wilcoxon signed-rank test on paired pre-pandemic and pandemic values; *r* as in Equation (2). Implausible reports (>16 h/day) excluded in either period. GPAQ: Global Physical Activity Questionnaire.

**Table 3:** Cronbach’s *α* for the AEBQ subscales by cohort, with 95% confidence intervals and the values reported in the original validation.

Subscale	Items	Students *α* (95% CI) (*n*)	Staff *α* (95% CI) (*n*)	Combined *α* (95% CI) (*n*)	Original Validation *α*
Enjoyment of Food (EF)	3	0.805 (0.777–0.831) (588)	0.802 (0.728–0.858) (112)	0.813 (0.788–0.836) (700)	0.84
Emotional Over-Eating (EOE)	5	0.905 (0.892–0.917) (581)	0.901 (0.868–0.927) (111)	0.905 (0.894–0.916) (692)	0.89
Emotional Under-Eating (EUE) ^1^	4	0.871 (0.853–0.887) (576)	0.853 (0.802–0.893) (110)	0.869 (0.852–0.884) (686)	0.85
Food Fussiness (FF)	5	0.684 (0.641–0.723) † (575)	0.643 (0.526–0.739) † (110)	0.675 (0.635–0.712) † (685)	0.73
Food Responsiveness (FR)	4	0.662 (0.615–0.705) † (581)	0.725 (0.630–0.800) (109)	0.673 (0.632–0.711) † (690)	0.79
Slowness in Eating (SE)	4	0.813 (0.787–0.837) (575)	0.769 (0.688–0.833) (106)	0.808 (0.784–0.831) (681)	0.65
Hunger (H)	5	0.700 (0.659–0.737) (570)	0.749 (0.666–0.817) (109)	0.707 (0.671–0.741) (679)	0.78
Satiety Responsiveness (SR)	4	0.641 (0.591–0.687) † (576)	0.571 (0.423–0.688) † (110)	0.633 (0.586–0.676) † (686)	0.80
EUE, five items including the duplicate ^1^	5	0.899 (0.885–0.911) (574)	0.898 (0.864–0.925) (110)	0.898 (0.886–0.910) (684)	0.85

Note. α: Cronbach’s alpha; CI: Feldt confidence interval; *n*: respondents with complete items for the subscale. Original validation: Hunot et al. [22]. † *α* < 0.70. ^1^ EUE was scored on four unique items because one item was rendered with the same Romanian wording as another item of the subscale; the five-item value is shown for transparency only. EUE: Emotional Under-Eating; AEBQ: Adult Eating Behaviour Questionnaire.

**Table 4:** Fit of the eight-factor AEBQ model by sample and estimator.

Sample	Estimator	*n*	χ^2^	*df*	CFI	TLI	RMSEA
Combined	DWLS	621	1507.9	499	0.911	0.900	0.057
Combined	ML	621	1700.1	499	0.863	0.846	0.062
Students	DWLS	520	1306.6	499	0.915	0.904	0.056
Students	ML	520	1505.3	499	0.863	0.846	0.062
Staff	DWLS	101	459.8	499	1.000	1.023	0.000
Staff	ML	101	868.9	499	0.772	0.743	0.086
Male	DWLS	340	1088.3	499	0.915	0.904	0.059
Male	ML	340	1299.3	499	0.844	0.824	0.069
Female	DWLS	281	680.1	499	0.963	0.959	0.036
Female	ML	281	1046.0	499	0.854	0.835	0.063

Note. Confirmatory factor analysis (CFA) on the 34 retained items (EUE scored on four items), complete cases only. DWLS: diagonally weighted least squares (primary, appropriate for ordinal indicators); ML: maximum likelihood (reference); CFI: comparative fit index; TLI: Tucker–Lewis index; RMSEA: root mean square error of approximation. All χ^2^ *p* < 0.001 except the staff DWLS model, for which χ^2^ < df (CFI bounded at 1; TLI above 1 by construction). The staff-only solution (*n* = 101 for 34 indicators) is overfitted and is shown for completeness only. EUE: Emotional Under-Eating; AEBQ: Adult Eating Behaviour Questionnaire.

**Table 5:** Mann–Whitney *U* tests for gender differences within cohorts.

Subscale	Men, Mean ± SD (*n*)	Women, Mean ± SD (*n*)	*U*	*p*	*r*	Effect Size
**Students**						
EF	3.78 ± 0.82 (339)	3.72 ± 0.81 (249)	44,265.5	0.307	0.042	Negligible
EOE	2.36 ± 0.90 (341)	2.74 ± 1.00 (249)	33,064.5	<0.001 **	0.189	Small
EUE	2.54 ± 0.89 (341)	2.93 ± 0.89 (248)	32,191.0	<0.001 **	0.204	Small
FF	2.45 ± 0.69 (339)	2.53 ± 0.65 (249)	39,145.0	0.131	0.062	Negligible
FR	2.86 ± 0.75 (341)	2.89 ± 0.66 (248)	41,430.5	0.674	0.017	Negligible
SE	2.58 ± 0.87 (340)	2.87 ± 0.87 (248)	34,094.0	<0.001 **	0.164	Small
H	2.76 ± 0.75 (340)	3.07 ± 0.72 (247)	31,701.5	<0.001 **	0.209	Small
SR	2.49 ± 0.66 (341)	2.87 ± 0.66 (247)	28,429.0	<0.001 **	0.278	Small
**Staff**						
EF	3.28 ± 0.70 (41)	3.31 ± 0.81 (71)	1426.0	0.860	0.017	Negligible
EOE	2.65 ± 0.94 (40)	2.86 ± 0.84 (72)	1281.0	0.334	0.091	Negligible
EUE	2.61 ± 0.62 (40)	2.83 ± 0.80 (72)	1261.5	0.274	0.102	Small
FF	2.66 ± 0.54 (40)	2.38 ± 0.60 (72)	1821.5	0.020	0.219	Small
FR	2.65 ± 0.77 (40)	2.73 ± 0.60 (72)	1391.5	0.768	0.028	Negligible
SE	2.72 ± 0.66 (40)	2.79 ± 0.76 (72)	1367.0	0.657	0.042	Negligible
H	2.70 ± 0.73 (40)	2.86 ± 0.72 (72)	1290.0	0.362	0.086	Negligible
SR	2.77 ± 0.63 (40)	2.86 ± 0.57 (72)	1297.5	0.384	0.082	Negligible

Note. SD: standard deviation; *U*: Mann–Whitney statistic; *r* = *Z*/√*N*. ** *p* < 0.00625 (Bonferroni-corrected threshold for eight subscales). Effect sizes: negligible *r* < 0.10; small 0.10–0.29; medium 0.30–0.49; large ≥ 0.50. EF: Enjoyment of Food; EOE: Emotional Over-Eating; EUE: Emotional Under-Eating; FF: Food Fussiness; FR: Food Responsiveness; SE: Slowness in Eating; H: Hunger; SR: Satiety Responsiveness.

**Table 6:** Mann–Whitney *U* tests for student–staff differences.

Subscale	Students, Mean ± SD (*n*)	Staff, Mean ± SD (*n*)	*U*	*p*	*r*	Effect Size
EF	3.76 ± 0.82 (588)	3.29 ± 0.77 (112)	44,113.0	<0.001 **	0.216	Small
EOE	2.52 ± 0.96 (590)	2.79 ± 0.88 (112)	27,238.0	0.003 **	0.111	Small
EUE	2.70 ± 0.91 (589)	2.75 ± 0.75 (112)	32,114.0	0.656	0.017	Negligible
FF	2.48 ± 0.67 (588)	2.48 ± 0.59 (112)	33,453.0	0.788	0.010	Negligible
FR	2.87 ± 0.71 (589)	2.70 ± 0.66 (112)	37,125.0	0.034	0.080	Negligible
SE	2.70 ± 0.88 (588)	2.77 ± 0.72 (112)	30,722.5	0.259	0.043	Negligible
H	2.89 ± 0.75 (587)	2.81 ± 0.72 (112)	34,708.0	0.347	0.035	Negligible
SR	2.65 ± 0.69 (588)	2.83 ± 0.59 (112)	27,817.5	0.009	0.098	Negligible

Note. SD: standard deviation; *U*: Mann–Whitney statistic; *r* = *Z*/√*N*. ** *p* < 0.00625 (Bonferroni-corrected threshold for eight subscales). Effect sizes as in Table 5. EF: Enjoyment of Food; EOE: Emotional Over-Eating; EUE: Emotional Under-Eating; FF: Food Fussiness; FR: Food Responsiveness; SE: Slowness in Eating; H: Hunger; SR: Satiety Responsiveness.

**Table 7:** Kruskal–Wallis tests for AEBQ subscale scores by COVID-19 infection status.

Subscale	Confirmed, Mean ± SD (*n*)	Not Confirmed, Mean ± SD (*n*)	Unknown, Mean ± SD (*n*)	*H*	*p*	*η* ^2^
**Students**						
EF	3.80 ± 0.89 (180)	3.77 ± 0.78 (263)	3.67 ± 0.80 (144)	2.94	0.230	0.002
EOE	2.60 ± 1.00 (184)	2.46 ± 0.88 (260)	2.53 ± 1.05 (145)	1.52	0.467	<0.001
EUE	2.78 ± 0.91 (184)	2.69 ± 0.88 (260)	2.66 ± 0.94 (144)	1.73	0.421	<0.001
FF	2.48 ± 0.72 (184)	2.50 ± 0.62 (259)	2.44 ± 0.70 (144)	1.14	0.565	<0.001
FR	2.94 ± 0.75 (183)	2.85 ± 0.69 (260)	2.82 ± 0.71 (145)	1.64	0.440	<0.001
SE	2.83 ± 0.89 (182)	2.62 ± 0.86 (259)	2.68 ± 0.90 (146)	6.69	0.035	0.008
H	3.05 ± 0.79 (182)	2.77 ± 0.73 (260)	2.92 ± 0.71 (144)	13.43	0.001 **	0.020
SR	2.68 ± 0.69 (182)	2.63 ± 0.69 (260)	2.66 ± 0.69 (145)	1.01	0.604	<0.001
**Staff**						
EF	3.33 ± 0.73 (53)	3.30 ± 0.83 (45)	3.17 ± 0.76 (14)	1.00	0.606	<0.001
EOE	2.82 ± 0.86 (53)	2.68 ± 0.92 (45)	3.00 ± 0.84 (14)	1.67	0.434	<0.001
EUE	2.81 ± 0.69 (53)	2.63 ± 0.80 (45)	2.88 ± 0.81 (14)	1.96	0.375	<0.001
FF	2.50 ± 0.59 (53)	2.50 ± 0.62 (45)	2.34 ± 0.53 (14)	1.06	0.589	<0.001
FR	2.76 ± 0.60 (53)	2.64 ± 0.75 (45)	2.70 ± 0.64 (14)	0.59	0.743	<0.001
SE	2.83 ± 0.71 (53)	2.58 ± 0.67 (45)	3.10 ± 0.82 (14)	5.08	0.079	0.028
H	2.85 ± 0.62 (53)	2.63 ± 0.79 (45)	3.20 ± 0.74 (14)	6.22	0.045	0.039
SR	2.83 ± 0.52 (53)	2.77 ± 0.68 (45)	3.00 ± 0.55 (14)	1.99	0.370	<0.001

Note. SD: standard deviation; *H*: Kruskal–Wallis statistic; *η*^2^: eta-squared, computed as in Equation (3); values are negative by construction when *H* < *k* − 1 and are shown as <0.001. ** *p* < 0.00625 (Bonferroni-corrected threshold for eight subscales). EF: Enjoyment of Food; EOE: Emotional Over-Eating; EUE: Emotional Under-Eating; FF: Food Fussiness; FR: Food Responsiveness; SE: Slowness in Eating; H: Hunger; SR: Satiety Responsiveness. AEBQ: Adult Eating Behaviour Questionnaire.

**Table 8:** Spearman correlations between AEBQ subscale scores and physical activity, perceived health and mental health, by cohort.

Correlate	EF	EOE	EUE	FF	FR	SE	H	SR
**Students** (*n* = 497–590)								
Physical activity, pandemic (MET-min/week)	0.057	−0.162 **	−0.027	−0.071	−0.017	−0.042	−0.106 *	−0.088 *
Physical activity, pre-pandemic	0.078	−0.046	−0.035	−0.060	0.040	−0.070	−0.025	−0.110 *
Change in physical activity	−0.060	−0.091 *	−0.008	0.036	−0.077	0.042	−0.084	0.009
Self-rated health, current	0.107 *	−0.115 **	−0.077	−0.057	−0.098 *	0.000	−0.169 **	−0.072
Health compared with pre-pandemic	0.068	−0.059	−0.022	0.006	−0.056	0.026	−0.043	−0.013
Mental health score	0.056	−0.222 **	−0.153 **	−0.116 **	−0.115 **	0.058	−0.306 **	−0.145 **
Emotional role limitations	−0.022	0.226 **	0.101 *	0.022	0.107 **	−0.014	0.191 **	0.109 **
Physical limitations	0.048	0.145 **	0.107 **	−0.001	0.089 *	0.037	0.131 **	0.060
Social interference	−0.034	0.169 **	0.129 **	0.023	0.063	0.010	0.223 **	0.076
Pain impact	−0.065	0.160 **	0.097 *	0.139 **	0.072	−0.003	0.239 **	0.150 **
Health-perception score	0.156 **	−0.138 **	−0.133 **	−0.150 **	−0.059	0.047	−0.247 **	−0.157 **
**Staff** (*n* = 95–112)								
Physical activity, pandemic (MET-min/week)	−0.205 *	−0.230 *	0.068	0.065	−0.022	0.050	−0.063	−0.052
Physical activity, pre-pandemic	−0.067	−0.154	0.147	0.001	−0.039	0.209 *	0.079	0.021
Change in physical activity	−0.069	−0.133	−0.222 *	0.009	−0.036	−0.317 **	−0.193	−0.220 *
Self-rated health, current	0.059	−0.276 **	0.101	0.007	−0.096	−0.215 *	−0.180	−0.210 *
Health compared with pre-pandemic	0.038	0.085	−0.202 *	−0.048	−0.085	−0.065	−0.075	−0.183
Mental health score	0.030	−0.189 *	−0.229 *	0.112	−0.316 **	−0.104	−0.358 **	−0.217 *
Emotional role limitations	0.093	0.218 *	0.090	−0.185	0.314 **	0.119	0.323 **	0.063
Physical limitations	0.000	0.177	0.132	−0.186	0.187	−0.006	0.266 **	0.093
Social interference	−0.012	0.196 *	0.123	−0.262 **	0.234 *	−0.075	0.328 **	0.043
Pain impact	0.083	0.234 *	0.090	−0.125	0.412 **	0.164	0.261 **	0.170
Health-perception score	0.031	−0.159	−0.087	−0.165	−0.176	−0.138	−0.196 *	−0.130

Note. Spearman’s *ρ*; pairwise deletion, with *n* range per cohort in the row header. * *p* < 0.05; ** *p* < 0.01 (unadjusted; Benjamini–Hochberg *q* values in Table S8). Physical activity as moderate-to-vigorous MET-min/week (GPAQ scoring; implausible reports excluded); change = pandemic − pre-pandemic. Self-rated health 1–5; health compared with pre-pandemic: 1 = much worse to 5 = much better; mental health score 1–6 (higher = better); limitations, social interference and pain: higher = worse; health-perception score: higher = better. EF: Enjoyment of Food; EOE: Emotional Over-Eating; EUE: Emotional Under-Eating; FF: Food Fussiness; FR: Food Responsiveness; SE: Slowness in Eating; H: Hunger; SR: Satiety Responsiveness; GPAQ: Global Physical Activity Questionnaire; MET: metabolic equivalent of task; AEBQ: Adult Eating Behaviour Questionnaire.

**Table 9:** Ordinary least squares regression of the Emotional Over-Eating score.

Predictor	Model 1: *B* (95% CI)	*p*	Model 2: *B* (95% CI)	*p*
Intercept	2.759 (2.278 to 3.241)	<0.001	2.399 (1.671 to 3.127)	<0.001
Female gender	0.243 (0.085 to 0.400)	0.003	0.301 (0.147 to 0.454)	<0.001
Staff cohort	0.198 (−0.007 to 0.403)	0.058	0.306 (0.110 to 0.502)	0.002
Confirmed COVID-19 infection	0.058 (−0.101 to 0.216)	0.476	0.051 (−0.101 to 0.204)	0.510
Physical activity, pandemic (log-transformed, standardised)	−0.083 (−0.161 to −0.005)	0.037	−0.078 (−0.154 to −0.002)	0.044
Change in physical activity (standardised)	−0.037 (−0.106 to 0.032)	0.298	−0.022 (−0.091 to 0.048)	0.543
Health compared with pre-pandemic	−0.033 (−0.109 to 0.042)	0.386	−0.045 (−0.117 to 0.027)	0.219
Mental health score	−0.118 (−0.211 to −0.026)	0.012	−0.134 (−0.226 to −0.042)	0.004
Emotional role limitations	0.129 (0.059 to 0.200)	<0.001	0.143 (0.074 to 0.212)	<0.001
Enjoyment of Food (EF)	—		0.240 (0.145 to 0.336)	<0.001
Emotional Under-Eating (EUE)	—		−0.180 (−0.288 to −0.073)	<0.001
Model fit	*n* = 590; *R*^2^ = 0.127; adj. *R*^2^ = 0.115; *F*(8, 581) = 10.17, *p* < 0.001		*n* = 575; *R*^2^ = 0.195; adj. *R*^2^ = 0.180; *F*(10, 564) = 12.99, *p* < 0.001	

Note. *B*: unstandardised coefficient with 95% confidence interval based on HC3 heteroscedasticity-consistent standard errors. Physical activity entered as log-transformed MET-min/week and change in physical activity as pandemic minus pre-pandemic MET-min/week, both standardised (*z*-scores); health compared with pre-pandemic: 1 = much worse to 5 = much better; mental health score 1–6 (higher = better); emotional role limitations 0–3. Model 1: primary model, predictors external to the AEBQ; Model 2: sensitivity analysis adding EF and EUE. Age group was examined separately (Section 2.4). A model adding current self-rated health (missing for 11% of respondents) is given in Table S6. EF: Enjoyment of Food; EUE: Emotional Under-Eating; MET: metabolic equivalent of task; AEBQ: Adult Eating Behaviour Questionnaire.

**Table 10:** Binary logistic regression of high Emotional Over-Eating (score ≥ 3.5).

Predictor	Model 1: OR (95% CI)	*p*	Model 2: OR (95% CI)	*p*
Female gender	2.18 (1.35–3.52)	0.002	3.35 (1.95–5.75)	<0.001
Staff cohort	1.42 (0.78–2.59)	0.255	1.81 (0.93–3.54)	0.081
Confirmed COVID-19 infection	0.98 (0.61–1.58)	0.931	0.98 (0.58–1.65)	0.939
Physical activity, pandemic (log-transformed, standardised)	0.82 (0.66–1.02)	0.072	0.85 (0.67–1.07)	0.160
Change in physical activity (standardised)	0.96 (0.76–1.21)	0.736	0.91 (0.70–1.19)	0.502
Health compared with pre-pandemic	0.90 (0.73–1.09)	0.281	0.94 (0.75–1.17)	0.552
Mental health score	0.76 (0.59–0.96)	0.024	0.65 (0.50–0.84)	0.001
Emotional role limitations	1.39 (1.11–1.74)	0.004	1.43 (1.12–1.82)	0.004
Enjoyment of Food (EF)	—		1.47 (1.06–2.04)	0.019
Emotional Under-Eating (EUE)	—		0.34 (0.25–0.47)	<0.001
Model fit	*n* = 590; events = 102; Nagelkerke *R*^2^ = 0.150; LR *χ*^2^(8) = 55.94, *p* < 0.001		*n* = 575; events = 102; Nagelkerke *R*^2^ = 0.307; LR *χ*^2^(10) = 118.86, *p* < 0.001	

Note. OR: odds ratio with 95% confidence interval; predictors coded as in Table 9. Model 1: primary model; Model 2: sensitivity analysis adding EF and EUE. Nagelkerke *R*^2^ and likelihood-ratio χ^2^ (LR χ^2^) given in the last row. The 15 respondents excluded from Model 2 for missing EF or EUE included no cases of high EOE, so both models rest on the same 102 events. EF: Enjoyment of Food; EUE: Emotional Under-Eating.

## Data Availability

The de-identified dataset (participant-level responses and derived variables, with a variable dictionary) and the analysis code (Python) are provided as File S1; the Romanian and English wording of every AEBQ item is given in Table S2, and the complete Romanian questionnaire is available from the corresponding author on request.

## Supplementary Materials

The following supporting information can be downloaded at: https://www.mdpi.com/article/10.3390/nu18213688/s1, Table S1: Flow of valid responses per instrument (upper block) and AEBQ subscale descriptive statistics by cohort and gender (lower block); Table S2: Item-level analysis of the Romanian AEBQ (original English and Romanian item wording, means, item–rest correlations) and Cronbach’s *α* by gender; Table S3: Standardised factor loadings of the eight-factor AEBQ model (diagonally weighted least squares, combined sample, *n* = 621); Table S4: Kruskal–Wallis tests for AEBQ subscales by physical-activity category (pandemic period; upper block) and distribution of the health-comparison item by group (lower block); Table S5: Spearman correlation matrix for the AEBQ subscales in the combined sample (pairwise *n* 683–701); Table S6: Additional regression models: OLS Model 1b (adding current self-rated health) for EOE and Models 1, 1b and 2 for SR and H (upper block); logistic Model 1b for high EOE (lower block); Table S7: Measurement invariance of the eight-factor AEBQ model across gender and across cohort (complete AEBQ data, *n* = 621); Table S8: Benjamini–Hochberg false discovery rate (FDR) adjustment of the exploratory Spearman correlations: correlations of the AEBQ subscales with physical activity, perceived health and mental health by cohort (upper block, Table 8 of the manuscript) and subscale intercorrelations (lower block, Figure 4 and Table S5); Table S9: Sensitivity analyses of the EOE models: equivalence tests with alternative margins (block a), logistic models of high EOE at alternative thresholds (block b) and Model 1 with alternative scoring of the emotional role limitations count (blocks c, OLS, and d, logistic, high EOE ≥ 3.5); Table S10: Age and cohort: AEBQ subscale scores of students and staff in the age-overlapping subgroup (block a), association of age group with the EOE, EF, SR and H scores within each cohort (block b) and the adjusted cohort coefficient for EOE in the age-overlapping subgroup (block c); Figure S1: Emotional Over-Eating score against moderate-to-vigorous physical activity during the pandemic, by cohort; File S1: de-identified dataset with variable dictionary and analysis code (zip).

## Abbreviations

The following abbreviations are used in this manuscript:

AEBQ	Adult Eating Behaviour Questionnaire
CFA	Confirmatory factor analysis
CFI	Comparative fit index
CI	Confidence interval
DWLS	Diagonally weighted least squares
EF	Enjoyment of Food
EOE	Emotional Over-Eating
EUE	Emotional Under-Eating
FDR	False discovery rate
FF	Food Fussiness
FR	Food Responsiveness
GPAQ	Global Physical Activity Questionnaire
H	Hunger
HC3	Heteroscedasticity-consistent standard errors (type 3)
IQR	Interquartile range
LR	Likelihood ratio
MET	Metabolic equivalent of task
ML	Maximum likelihood
MLR	Robust maximum likelihood
OR	Odds ratio
RMSEA	Root mean square error of approximation
SD	Standard deviation
SE	Slowness in Eating
SF-36	36-item Short Form Health Survey
SR	Satiety Responsiveness
TLI	Tucker–Lewis index
TOST	Two one-sided tests
TUM	Technical University of Moldova
WHO	World Health Organization
WLSMV	Weighted least squares mean- and variance-adjusted
