# Supplementary material for: Gender and Psychological State, More than Pandemic-Related Lifestyle Change, Are Associated with Emotional Over-Eating Among University Students and Staff in Moldova
Source: Nutrients. 2026 Nov 7;18(21):3688. doi: 10.3390/nu18213688 (PMC13765520; doi:10.3390/nu18213688)
Supplement: Supplementary file 1 [file nutrients-18-03688-s001.zip › nutrients-4586275-supplementary.pdf]

Supplementary Materials

Gender and Psychological State, More than Pandemic-Related Lifestyle Change, Are Associated with Emotional Over-Eating Among University Students and Staff in Moldova

Rodica Siminiuc, Dinu Țurcanu and Sergiu Siminiuc

Contents: Table S1, flow of valid responses per instrument and AEBQ subscale descriptive statistics by cohort and gender; Table S2, item-level analysis of the Romanian AEBQ and Cronbach's α by gender; Table S3, standardised factor loadings of the eight-factor model; Table S4, Kruskal–Wallis tests by physical-activity category and distribution of the health-comparison item; Table S5, Spearman correlation matrix in the combined sample; Table S6, additional regression models; Table S7, measurement invariance of the eight-factor model across gender and cohort; Table S8, FDR-adjusted correlations; Table S9, sensitivity analyses of the EOE models (equivalence margins, thresholds, scoring of missing items); Table S10, age and cohort analyses; Figure S1, Emotional Over-Eating score against physical activity during the pandemic. File S1 (de-identified dataset, variable dictionary and analysis code) is provided separately.

Table S1. Flow of valid responses per instrument (upper block) and AEBQ subscale descriptive statistics by cohort and gender (lower block).

Step	Students	Staff
Responses received	623	121
AEBQ EF scored (≥75% items)	588	112
AEBQ EOE scored (≥75% items)	590	112
AEBQ EUE scored (≥75% items)	589	112
AEBQ FF scored (≥75% items)	588	112
AEBQ FR scored (≥75% items)	589	112
AEBQ SE scored (≥75% items)	588	112
AEBQ H scored (≥75% items)	587	112
AEBQ SR scored (≥75% items)	588	112
PA pandemic (cleaned)	572	108
PA pre-pandemic (cleaned)	541	104
PA paired	524	103
Self-rated health current	548	114
Health-comparison item	623	121
Mental health score	618	121
Emotional limitations	617	119
Regression Model 1 complete cases	497	93

Group	Subscale	n	Mean	SD	Median	Q1	Q3
Students Total	EF	588	3.760	0.820	3.670	3.330	4.330
Students Total	EOE	590	2.520	0.960	2.400	2.000	3.150
Students Total	EUE	589	2.700	0.910	2.750	2.000	3.250
Students Total	FF	588	2.480	0.670	2.500	2.000	3.000
Students Total	FR	589	2.870	0.710	3.000	2.500	3.250
Students Total	SE	588	2.700	0.880	2.750	2.000	3.250
Students Total	H	587	2.890	0.750	2.800	2.400	3.400
Students Total	SR	588	2.650	0.690	2.670	2.250	3.000
Students Male	EF	339	3.780	0.820	3.670	3.330	4.330
Students Male	EOE	341	2.360	0.900	2.200	1.800	3.000
Students Male	EUE	341	2.540	0.890	2.500	2.000	3.000

Group	Subscale	n	Mean	SD	Median	Q1	Q3
Students Male	FF	339	2.450	0.690	2.400	2.000	2.800
Students Male	FR	341	2.860	0.750	3.000	2.500	3.250
Students Male	SE	340	2.580	0.870	2.500	2.000	3.000
Students Male	H	340	2.760	0.750	2.800	2.200	3.200
Students Male	SR	341	2.490	0.660	2.500	2.000	3.000
Students Female	EF	249	3.720	0.810	3.670	3.330	4.330
Students Female	EOE	249	2.740	1.000	2.800	2.000	3.400
Students Female	EUE	248	2.930	0.890	3.000	2.250	3.500
Students Female	FF	249	2.530	0.650	2.600	2.200	3.000
Students Female	FR	248	2.890	0.660	2.880	2.500	3.250
Students Female	SE	248	2.870	0.870	2.750	2.250	3.500
Students Female	H	247	3.070	0.720	3.000	2.600	3.600
Students Female	SR	247	2.870	0.660	3.000	2.500	3.250
Staff Total	EF	112	3.290	0.770	3.330	2.670	3.670
Staff Total	EOE	112	2.790	0.880	2.800	2.200	3.400
Staff Total	EUE	112	2.750	0.750	2.750	2.250	3.000
Staff Total	FF	112	2.480	0.590	2.400	2.000	2.850
Staff Total	FR	112	2.700	0.660	2.750	2.250	3.000
Staff Total	SE	112	2.770	0.720	2.750	2.250	3.250
Staff Total	H	112	2.810	0.720	2.800	2.400	3.200
Staff Total	SR	112	2.830	0.590	2.880	2.500	3.250
Staff Male	EF	41	3.280	0.700	3.330	3.000	3.670
Staff Male	EOE	40	2.650	0.940	2.900	2.000	3.000
Staff Male	EUE	40	2.610	0.620	2.750	2.190	3.000
Staff Male	FF	40	2.660	0.540	2.700	2.200	3.000
Staff Male	FR	40	2.650	0.770	2.750	2.000	3.060
Staff Male	SE	40	2.720	0.660	2.750	2.190	3.000
Staff Male	H	40	2.700	0.730	2.800	2.400	3.200
Staff Male	SR	40	2.780	0.630	2.750	2.250	3.060
Staff Female	EF	71	3.310	0.810	3.330	2.670	4.000
Staff Female	EOE	72	2.860	0.840	2.800	2.200	3.600
Staff Female	EUE	72	2.830	0.800	3.000	2.250	3.060
Staff Female	FF	72	2.380	0.600	2.330	2.000	2.800
Staff Female	FR	72	2.730	0.600	2.750	2.440	3.000
Staff Female	SE	72	2.790	0.760	2.750	2.250	3.250
Staff Female	H	72	2.860	0.720	2.800	2.400	3.200
Staff Female	SR	72	2.860	0.570	3.000	2.500	3.250

Note. n: number of valid responses; SD: standard deviation; Q1, Q3: first and third quartiles. Subscale scores range from 1 to 5 (≥ 75% of items answered). AEBQ: Adult Eating Behaviour Questionnaire; EF: Enjoyment of Food; EOE: Emotional Over-Eating; EUE: Emotional Under-Eating; FF: Food Fussiness; FR: Food Responsiveness; SE: Slowness in Eating; H: Hunger; SR: Satiety Responsiveness.

Table S2. Item-level analysis of the Romanian AEBQ (original English and Romanian item wording, means, item–rest correlations) and Cronbach's α by gender.

Subscale	Item	Reverse-scored	Item text (EN, original AEBQ)	Item text (RO)	Cohort	n	Mean	SD	Item-rest r
EF	Q1	no	I love food	Ador mâncarea	Students	588	4.040	0.940	0.702
EF	Q2	no	I enjoy eating	Îmi place să mănânc	Students	588	4.030	0.900	0.772
EF	Q3	no	I look forward to mealtimes	Aștept cu nerăbdare ora mesei	Students	588	3.210	1.050	0.512
EF	Q1	no	I love food	Ador mâncarea	Staff	112	3.570	0.800	0.690
EF	Q2	no	I enjoy eating	Îmi place să mănânc	Staff	112	3.500	0.870	0.715
EF	Q3	no	I look forward to mealtimes	Aștept cu nerăbdare ora mesei	Staff	112	2.810	1.040	0.569
EOE	Q4	no	I eat more when I'm annoyed	Mănânc mai mult când sunt enervat	Students	581	2.690	1.250	0.751
EOE	Q5	no	I eat more when I'm worried	Mănânc mai mult când sunt îngrijorat	Students	581	2.510	1.140	0.801
EOE	Q6	no	I eat more when I'm upset	Mănânc mai mult când sunt supărat	Students	581	2.380	1.150	0.793
EOE	Q7	no	I eat more when I'm anxious	Mănânc mai mult când sunt anxios	Students	581	2.480	1.020	0.692
EOE	Q8	no	I eat more when I'm irritable	Mănânc mai mult când sunt nervos	Students	581	2.530	1.080	0.782
EOE	Q4	no	I eat more when I'm annoyed	Mănânc mai mult când sunt enervat	Staff	111	2.960	1.170	0.813
EOE	Q5	no	I eat more when I'm worried	Mănânc mai mult când sunt îngrijorat	Staff	111	2.780	1.080	0.808
EOE	Q6	no	I eat more when I'm upset	Mănânc mai mult când sunt supărat	Staff	111	2.710	1.060	0.836
EOE	Q7	no	I eat more when I'm anxious	Mănânc mai mult când sunt anxios	Staff	111	2.680	0.890	0.573
EOE	Q8	no	I eat more when I'm irritable	Mănânc mai mult când sunt nervos	Staff	111	2.810	1.000	0.749
EUE	Q9	no	I eat less when I'm worried	Mănânc mai puțin când sunt îngrijorat	Students	576	2.720	1.120	0.659
EUE	Q10	no	I eat less when I'm annoyed	Mănânc mai puțin când sunt enervat	Students	576	2.690	1.080	0.788
EUE	Q11	no	I eat less when I'm upset	Mănânc mai puțin când sunt supărat	Students	576	2.720	1.070	0.756
EUE	Q13	no	I eat less when I'm anxious	Mănânc mai puțin când sunt anxios	Students	576	2.690	1.030	0.699
EUE	Q9	no	I eat less when I'm worried	Mănânc mai puțin când sunt îngrijorat	Staff	110	2.790	0.920	0.754
EUE	Q10	no	I eat less when I'm annoyed	Mănânc mai puțin când sunt enervat	Staff	110	2.630	0.940	0.690
EUE	Q11	no	I eat less when I'm upset	Mănânc mai puțin când sunt supărat	Staff	110	2.800	0.950	0.792

Subscale	Item	Reverse-scored	Item text (EN, original AEBQ)	Item text (RO)	Cohort	n	Mean	SD	Item-rest r
EUE	Q13	no	I eat less when I'm anxious	Mănânc mai puțin când sunt anxios	Staff	110	2.750	0.790	0.552
FF	Q14	no	I often decide that I don't like a food, before tasting it	De multe ori decid că nu-mi place o mâncare înainte de a o gusta	Students	575	3.080	1.170	0.309
FF	Q15	no	I refuse new foods at first	Refuz la început alimentele noi	Students	575	2.410	1.000	0.425
FF	Q16	yes	I enjoy tasting new foods	Îmi place să gust alimente noi	Students	575	2.340	0.960	0.579
FF	Q17	yes	I am interested in tasting food I haven't tasted before	Sunt interesat să gust mâncare nouă pe care nu am mai gustat-o până acum	Students	575	2.340	0.980	0.520
FF	Q18	yes	I enjoy eating a wide variety of foods	Îmi plac o gamă diversificată de alimente	Students	575	2.250	0.930	0.397
FF	Q14	no	I often decide that I don't like a food, before tasting it	De multe ori decid că nu-mi place o mâncare înainte de a o gusta	Staff	110	2.730	1.000	0.171
FF	Q15	no	I refuse new foods at first	Refuz la început alimentele noi	Staff	110	2.510	0.920	0.505
FF	Q16	yes	I enjoy tasting new foods	Îmi place să gust alimente noi	Staff	110	2.410	0.930	0.521
FF	Q17	yes	I am interested in tasting food I haven't tasted before	Sunt interesat să gust mâncare nouă pe care nu am mai gustat-o până acum	Staff	110	2.610	0.950	0.422
FF	Q18	yes	I enjoy eating a wide variety of foods	Îmi plac o gamă diversificată de alimente	Staff	110	2.180	0.810	0.407
FR	Q19	no	I often feel hungry when I am with someone who is eating	De multe ori mi se face foame când sunt cu cineva care mănâncă	Students	581	3.110	1.120	0.494
FR	Q20	no	Given the choice, I would eat most of the time	Dacă aș putea alege, aș mânca de cele mai multe ori	Students	581	2.590	1.030	0.419
FR	Q21	no	I am always thinking about food	Mă gândesc întotdeauna la mâncare	Students	581	2.210	0.950	0.476
FR	Q22	no	When I see or smell food that I like, it makes me want to eat	Când văd sau miros alimente care îmi plac, mă face să vreau să mănânc	Students	581	3.570	0.950	0.388
FR	Q19	no	I often feel hungry when I am with	De multe ori mi se face foame când	Staff	109	3.030	0.900	0.575

Subscale	Item	Reverse-scored	Item text (EN, original AEBQ)	Item text (RO)	Cohort	n	Mean	SD	Item-rest r
			someone who is eating	sunt cu cineva care mănâncă					
FR	Q20	no	Given the choice, I would eat most of the time	Dacă aș putea alege, aș mânca de cele mai multe ori	Staff	109	2.350	0.950	0.512
FR	Q21	no	I am always thinking about food	Mă gândesc întotdeauna la mâncare	Staff	109	2.000	0.780	0.473
FR	Q22	no	When I see or smell food that I like, it makes me want to eat	Când văd sau miros alimente care îmi plac, mă face să vreau să mănânc	Staff	109	3.390	0.960	0.505
SE	Q23	no	I am often last at finishing my meals	De multe ori termin ultimul masa	Students	575	2.770	1.130	0.699
SE	Q24	no	I eat more and more slowly during the course of a meal	Mănânc din ce în ce mai încet în timpul mesei	Students	575	2.620	0.990	0.563
SE	Q25	no	I eat slowly	Mănânc încet	Students	575	2.820	1.100	0.749
SE	Q26	yes	I often finish my meals quickly	De multe ori termin repede să mănânc	Students	575	2.630	1.190	0.533
SE	Q23	no	I am often last at finishing my meals	De multe ori termin ultimul masa	Staff	106	2.880	0.930	0.562
SE	Q24	no	I eat more and more slowly during the course of a meal	Mănânc din ce în ce mai încet în timpul mesei	Staff	106	2.650	0.810	0.560
SE	Q25	no	I eat slowly	Mănânc încet	Staff	106	2.900	0.960	0.767
SE	Q26	yes	I often finish my meals quickly	De multe ori termin repede să mănânc	Staff	106	2.700	1.040	0.431
H	Q27	no	I often notice my stomach rumbling	De multe ori observ stomacul meu produce sunete	Students	570	2.930	1.100	0.405
H	Q28	no	If I miss a meal I get irritable	Dacă sar peste ora mesei, mă irit	Students	570	2.610	1.110	0.446
H	Q29	no	I often feel so hungry that I have to eat something right away	De multe ori mă simt atât de flămând încât trebuie să mănânc ceva imediat	Students	570	3.080	1.120	0.540
H	Q30	no	I often feel hungry	De multe ori simt foame	Students	570	3.070	0.980	0.526
H	Q31	no	If I miss a meal I get light-headed	Dacă nu mănânc la timp am durei de cap	Students	570	2.770	1.220	0.382
H	Q27	no	I often notice my stomach rumbling	De multe ori observ stomacul meu produce sunete	Staff	109	2.710	1.040	0.404
H	Q28	no	If I miss a meal I get irritable	Dacă sar peste ora mesei, mă irit	Staff	109	2.620	1.020	0.625

Subscale	Item	Reverse-scored	Item text (EN, original AEBQ)	Item text (RO)	Cohort	n	Mean	SD	Item-rest r
H	Q29	no	I often feel so hungry that I have to eat something right away	De multe ori mă simt atât de flămând încât trebuie să mănânc ceva imediat	Staff	109	3.000	1.020	0.587
H	Q30	no	I often feel hungry	De multe ori simt foame	Staff	109	2.840	0.940	0.542
H	Q31	no	If I miss a meal I get light-headed	Dacă nu mănânc la timp am durei de cap	Staff	109	2.810	1.060	0.430
SR	Q32	no	I often leave food on my plate at the end of a meal	De multe ori las mâncare pe farfurie la sfârșitul mesei	Students	576	2.320	1.100	0.400
SR	Q33	no	I often get full before my meal is finished	De multe ori mă simt sătul înainte ca să termin de mâncat	Students	576	2.970	1.000	0.441
SR	Q34	no	I cannot eat a meal if I have had a snack just before	Nu pot mânca o masă dacă am luat o gustare chiar înainte	Students	576	2.510	0.940	0.373
SR	Q35	no	I get full up easily	Mă simt rapid sătul	Students	576	2.810	0.920	0.481
SR	Q32	no	I often leave food on my plate at the end of a meal	De multe ori las mâncare pe farfurie la sfârșitul mesei	Staff	110	2.460	1.030	0.357
SR	Q33	no	I often get full before my meal is finished	De multe ori mă simt sătul înainte ca să termin de mâncat	Staff	110	3.070	0.950	0.358
SR	Q34	no	I cannot eat a meal if I have had a snack just before	Nu pot mânca o masă dacă am luat o gustare chiar înainte	Staff	110	2.950	0.820	0.283
SR	Q35	no	I get full up easily	Mă simt rapid sătul	Staff	110	2.830	0.790	0.436

Subscale	k items	Students Male α (95% CI)	Students Male n	Students Female α (95% CI)	Students Female n	Staff Male α (95% CI)	Staff Male n	Staff Female α (95% CI)	Staff Female n
EF	3	0.801 (0.761–0.835)	339	0.812 (0.767–0.849)	249	0.739 (0.562–0.852)	41	0.828 (0.745–0.887)	71
EOE	5	0.900 (0.882–0.916)	337	0.903 (0.883–0.921)	244	0.946 (0.914–0.969)	39	0.870 (0.816–0.912)	72
EUE	4	0.879 (0.856–0.899)	334	0.846 (0.812–0.876)	242	0.855 (0.763–0.917)	39	0.849 (0.782–0.899)	71
FF	5	0.669 (0.609–0.722)	332	0.709 (0.646–0.763)	243	0.554 (0.291–0.740)	40	0.669 (0.528–0.777)	70
FR	4	0.668 (0.607–	338	0.653 (0.576–	243	0.838 (0.737–	40	0.635 (0.471–	69

Subscale	k items	Students Male α (95% CI)	Students Male n	Students Female α (95% CI)	Students Female n	Staff Male α (95% CI)	Staff Male n	Staff Female α (95% CI)	Staff Female n
		0.723)		0.720)		0.907)		0.758)	
SE	4	0.801 (0.764–0.834)	332	0.817 (0.777–0.852)	243	0.777 (0.638–0.872)	40	0.767 (0.659–0.847)	66
H	5	0.696 (0.641–0.746)	327	0.680 (0.611–0.739)	243	0.793 (0.668–0.880)	39	0.724 (0.607–0.814)	70
SR	4	0.595 (0.519–0.662)	334	0.640 (0.559–0.708)	242	0.633 (0.404–0.789)	40	0.529 (0.319–0.686)	70
EUE (5 items incl. duplicate Q12)	5	0.911 (0.895–0.925)	332	0.873 (0.846–0.896)	242	0.893 (0.829–0.938)	39	0.897 (0.853–0.930)	71
SE with Q23 reverse-keyed (keying check; not used)	4	-0.338 (-0.589–-0.117)	332	-0.186 (-0.450–0.040)	243	-0.612 (-1.617–0.072)	40	0.197 (-0.174–0.472)	66

Note. Item–rest *r*: correlation between the item and the sum of the remaining items of its subscale; reverse-keyed items are scored 6 − response. Q12 was administered with the same Romanian wording as Q10 and is excluded from the EUE score. Romanian item wording is reproduced as administered. α: Cronbach's alpha with Feldt 95% confidence interval; *n*: respondents with complete items. AEBQ: Adult Eating Behaviour Questionnaire; EF: Enjoyment of Food; EOE: Emotional Over-Eating; EUE: Emotional Under-Eating; FF: Food Fussiness; FR: Food Responsiveness; SE: Slowness in Eating; H: Hunger; SR: Satiety Responsiveness.

Table S3. Standardised factor loadings of the eight-factor AEBQ model (diagonally weighted least squares, combined sample, n = 621).

Factor	Item	Std. loading	p	Item text (EN, original AEBQ)	Item text (RO)
EF	Q1	0.689	-	I love food	Ador mâncarea
EF	Q2	0.779	< 0.001	I enjoy eating	Îmi place să mănânc
EF	Q3	0.819	< 0.001	I look forward to mealtimes	Aștept cu nerăbdare ora mesei
EOE	Q4	0.824	-	I eat more when I'm annoyed	Mănânc mai mult când sunt enervat
EOE	Q5	0.863	< 0.001	I eat more when I'm worried	Mănânc mai mult când sunt îngrijorat
EOE	Q6	0.904	< 0.001	I eat more when I'm upset	Mănânc mai mult când sunt supărat
EOE	Q7	0.721	< 0.001	I eat more when I'm anxious	Mănânc mai mult când sunt anxios
EOE	Q8	0.806	< 0.001	I eat more when I'm irritable	Mănânc mai mult când sunt nervos
EUE	Q9	0.732	-	I eat less when I'm worried	Mănânc mai puțin când sunt îngrijorat
EUE	Q10	0.841	< 0.001	I eat less when I'm annoyed	Mănânc mai puțin când sunt enervat
EUE	Q11	0.847	< 0.001	I eat less when I'm upset	Mănânc mai puțin când sunt supărat
EUE	Q13	0.763	< 0.001	I eat less when I'm anxious	Mănânc mai puțin când sunt anxios
FF	Q14	0.162	-	I often decide that I don't like a food, before tasting it	De multe ori decid că nu-mi place o mâncare înainte de a o gusta

Factor	Item	Std. loading	p	Item text (EN, original AEBQ)	Item text (RO)
FF	Q15	0.269	0.001	I refuse new foods at first	Refuz la început alimentele noi
FF	Q16	0.764	< 0.001	I enjoy tasting new foods	Îmi place să gust alimente noi
FF	Q17	0.861	< 0.001	I am interested in tasting food I haven’t tasted before	Sunt interesat să gust mâncare nouă pe care nu am mai gustat-o până acum
FF	Q18	0.546	< 0.001	I enjoy eating a wide variety of foods	Îmi plac o gamă diversificată de alimente
FR	Q19	0.583	-	I often feel hungry when I am with someone who is eating	De multe ori mi se face foame când sunt cu cineva care mănâncă
FR	Q20	0.588	< 0.001	Given the choice, I would eat most of the time	Dacă aș putea alege, aș mânca de cele mai multe ori
FR	Q21	0.659	< 0.001	I am always thinking about food	Mă gândesc întotdeauna la mâncare
FR	Q22	0.500	< 0.001	When I see or smell food that I like, it makes me want to eat	Când văd sau miros alimente care îmi plac, mă face să vreau să mănânc
SE	Q23	0.785	-	I am often last at finishing my meals	De multe ori termin ultimul masa
SE	Q24	0.758	< 0.001	I eat more and more slowly during the course of a meal	Mănânc din ce în ce mai încet în timpul mesei
SE	Q25	0.837	< 0.001	I eat slowly	Mănânc încet
SE	Q26	0.466	< 0.001	I often finish my meals quickly	De multe ori termin repede să mănânc
H	Q27	0.500	-	I often notice my stomach rumbling	De multe ori observ stomacul meu produce sunete
H	Q28	0.650	< 0.001	If I miss a meal I get irritable	Dacă sar peste ora mesei, mă irit
H	Q29	0.682	< 0.001	I often feel so hungry that I have to eat something right away	De multe ori mă simt atât de flămând încât trebuie să mănânc ceva imediat
H	Q30	0.615	< 0.001	I often feel hungry	De multe ori simt foame
H	Q31	0.431	< 0.001	If I miss a meal I get light-headed	Dacă nu mănânc la timp am durei de cap
SR	Q32	0.614	-	I often leave food on my plate at the end of a meal	De multe ori las mâncare pe farfurie la sfârșitul mesei
SR	Q33	0.543	< 0.001	I often get full before my meal is finished	De multe ori mă simt sătul înainte ca să termin de mâncat
SR	Q34	0.523	< 0.001	I cannot eat a meal if I have had a snack just before	Nu pot mânca o masă dacă am luat o gustare chiar înainte
SR	Q35	0.498	< 0.001	I get full up easily	Mă simt rapid sătul

Note. p: significance of the loading; “-” marks the reference indicator of each factor (loading fixed for identification). AEBQ: Adult Eating Behaviour Questionnaire; EF: Enjoyment of Food; EOE: Emotional Over-Eating; EUE: Emotional Under-Eating; FF: Food Fussiness; FR: Food Responsiveness; SE: Slowness in Eating; H: Hunger; SR: Satiety Responsiveness.

Table S4. Kruskal–Wallis tests for AEBQ subscales by physical-activity category (pandemic period; upper block) and distribution of the health-comparison item by group (lower block).

Cohort	Subscale	n	Inactive	Minimally active	Health-enhancing	H	p	Bonferroni (p < 0.00625)	η²
Students	EF	539	Inactive: 3.71 ± 0.79 (167)	Min. active: 3.79 ± 0.79 (217)	HEPA: 3.82 ± 0.84 (155)	2.210	0.331		0.000
Students	EOE	541	Inactive: 2.66 ± 1.01 (170)	Min. active: 2.59 ± 0.98 (214)	HEPA: 2.30 ± 0.88 (157)	11.230	0.004	**	0.017
Students	EUE	540	Inactive: 2.73 ± 0.95 (170)	Min. active: 2.75 ± 0.87 (213)	HEPA: 2.61 ± 0.94 (157)	1.900	0.388		0.000
Students	FF	539	Inactive: 2.56 ± 0.68 (170)	Min. active: 2.43 ± 0.68 (213)	HEPA: 2.43 ± 0.65 (156)	4.980	0.083		0.006
Students	FR	540	Inactive: 2.87 ± 0.74 (170)	Min. active: 2.93 ± 0.67 (213)	HEPA: 2.81 ± 0.70 (157)	1.470	0.480		0.000
Students	SE	539	Inactive: 2.74 ± 0.91 (170)	Min. active: 2.76 ± 0.88 (212)	HEPA: 2.64 ± 0.89 (157)	1.910	0.384		0.000
Students	H	538	Inactive: 2.96 ± 0.74 (169)	Min. active: 2.96 ± 0.71 (212)	HEPA: 2.77 ± 0.76 (157)	7.390	0.025		0.010
Students	SR	539	Inactive: 2.74 ± 0.68 (169)	Min. active: 2.67 ± 0.69 (212)	HEPA: 2.56 ± 0.68 (158)	5.010	0.082		0.006
Staff	EF	99	Inactive: 3.34 ± 0.93 (34)	Min. active: 3.42 ± 0.66 (37)	HEPA: 3.05 ± 0.53 (28)	4.720	0.095		0.028
Staff	EOE	100	Inactive: 2.92 ± 1.00 (35)	Min. active: 2.78 ± 0.83 (37)	HEPA: 2.51 ± 0.77 (28)	3.260	0.195		0.013
Staff	EUE	100	Inactive: 2.64 ± 0.77 (35)	Min. active: 2.80 ± 0.79 (37)	HEPA: 2.83 ± 0.77 (28)	0.660	0.718		0.000
Staff	FF	100	Inactive: 2.49 ± 0.77 (35)	Min. active: 2.41 ± 0.43 (37)	HEPA: 2.57 ± 0.50 (28)	1.450	0.483		0.000
Staff	FR	100	Inactive: 2.68 ± 0.70 (35)	Min. active: 2.74 ± 0.64 (37)	HEPA: 2.66 ± 0.72 (28)	0.070	0.965		0.000
Staff	SE	100	Inactive: 2.78 ± 0.88 (35)	Min. active: 2.77 ± 0.56 (37)	HEPA: 2.71 ± 0.68 (28)	0.100	0.951		0.000
Staff	H	100	Inactive: 2.79 ± 0.76 (35)	Min. active: 2.86 ± 0.80 (37)	HEPA: 2.71 ± 0.63 (28)	0.160	0.921		0.000
Staff	SR	100	Inactive: 2.84 ± 0.68 (35)	Min. active: 2.84 ± 0.51 (37)	HEPA: 2.80 ± 0.61 (28)	0.170	0.917		0.000

Group	n	Much worse	Somewhat worse	Same	Somewhat better	Much better	Worse (1–2) %	Better (4–5) %
Students	362	14 (3.9)	53 (14.6)	182 (50.3)	48 (13.3)	65 (18.0)	18.500	31.200

Group	n	Much worse	Somewhat worse	Same	Somewhat better	Much better	Worse (1–2) %	Better (4–5) %
Male Students	261	15 (5.7)	52 (19.9)	109 (41.8)	36 (13.8)	49 (18.8)	25.700	32.600
Female Students	623	29 (4.7)	105 (16.9)	291 (46.7)	84 (13.5)	114 (18.3)	21.500	31.800
Total								
Staff Male	42	2 (4.8)	11 (26.2)	21 (50.0)	3 (7.1)	5 (11.9)	31	19
Staff Female	79	5 (6.3)	27 (34.2)	28 (35.4)	6 (7.6)	13 (16.5)	40.500	24.100
Staff Total	121	7 (5.8)	38 (31.4)	49 (40.5)	9 (7.4)	18 (14.9)	37.200	22.300

Test	n
Cohort × change (worse/same/better): $\chi^2(2) = 14.21$, $p < 0.001$, Cramér V = 0.138	744
Gender × change, students: $\chi^2(2) = 6.06$, $p = 0.048$, Cramér V = 0.099	623
Gender × change, staff: $\chi^2(2) = 2.42$, $p = 0.299$, Cramér V = 0.141	121

Note. Physical-activity categories according to the GPAQ thresholds (inactive < 600, minimally active 600–3000, health-enhancing > 3000 MET-min/week; GPAQ: Global Physical Activity Questionnaire; MET: metabolic equivalent of task). H: Kruskal–Wallis statistic; η^2: eta-squared as in Equation (3) of the manuscript; Bonferroni-corrected threshold $p < 0.00625$. Health-comparison item: 1 = much worse to 5 = much better than before the pandemic; worse = 1–2, better = 4–5. χ^2 tests on the three-category variable with Cramér's V. AEBQ: Adult Eating Behaviour Questionnaire; EF: Enjoyment of Food; EOE: Emotional Over-Eating; EUE: Emotional Under-Eating; FF: Food Fussiness; FR: Food Responsiveness; SE: Slowness in Eating; H: Hunger; SR: Satiety Responsiveness.

Table S5. Spearman correlation matrix for the AEBQ subscales in the combined sample (pairwise *n* 683–701).

Subscale	EF	EOE	EUE	FF	FR	SE	H	SR
EF	—							
EOE	0.14**	—						
EUE	−0.06	−0.03	—					
FF	−0.24**	0.05	0.04	—				
FR	0.44**	0.37**	0.14**	−0.14**	—			
SE	−0.05	0.01	0.15**	−0.02	−0.01	—		
H	0.29**	0.44**	0.21**	−0.03	0.52**	0.05	—	
SR	−0.19**	0.14**	0.31**	0.15**	−0.03	0.24**	0.20**	—

Note. Spearman's ρ; ** $p < 0.01$; * $p < 0.05$. AEBQ: Adult Eating Behaviour Questionnaire; EF: Enjoyment of Food; EOE: Emotional Over-Eating; EUE: Emotional Under-Eating; FF: Food Fussiness; FR: Food Responsiveness; SE: Slowness in Eating; H: Hunger; SR: Satiety Responsiveness.

Table S6. Additional regression models: OLS Model 1b (adding current self-rated health) for EOE and Models 1, 1b and 2 for SR and H (upper block); logistic Model 1b for high EOE (lower block).

Model	Predictor	B	SE (HC3)	95% CI	p	VIF
EOE, Model 1b (+ self-rated health)	Intercept	2.701	0.269	2.173 to 3.229	< 0.001	
EOE, Model 1b (+ self-rated health)	Female gender	0.277	0.086	0.109 to 0.445	0.001	1.110
EOE, Model 1b (+ self-rated health)	Staff cohort	0.222	0.109	0.008 to 0.436	0.042	1.090
EOE, Model 1b (+ self-rated health)	Confirmed COVID-19 infection	0.050	0.086	−0.119 to 0.219	0.562	1.030
EOE, Model 1b (+ self-rated health)	Physical activity, pandemic (log-transformed, standardised)	−0.049	0.042	−0.131 to 0.034	0.248	1.080
EOE, Model 1b (+ self-rated health)	Change in physical activity (standardised)	−0.044	0.037	−0.117 to 0.028	0.227	1.080
EOE, Model 1b (+ self-rated health)	Health compared with pre-pandemic	−0.022	0.040	−0.101 to 0.058	0.591	1.070
EOE, Model 1b (+ self-rated health)	Mental health score	−0.124	0.052	−0.226 to −0.023	0.016	1.490
EOE, Model 1b (+ self-rated health)	Emotional role limitations	0.139	0.039	0.063 to 0.215	< 0.001	1.330
EOE, Model 1b (+ self-rated health)	Self-rated health, current	0.004	0.046	−0.087 to 0.095	0.936	1.330
EOE, Model 1b (+ self-rated health)	n = 525; $R^2 = 0.130$; adj. $R^2 = 0.115$; $F(9,515) = 8.03$, $p < 0.001$					
SR, Model 1	Intercept	2.703	0.183	2.344 to 3.061	< 0.001	
SR, Model 1	Female gender	0.329	0.060	0.212 to 0.446	< 0.001	1.110
SR, Model 1	Staff cohort	0.126	0.075	−0.021 to 0.274	0.093	1.070
SR, Model 1	Confirmed COVID-19 infection	0.009	0.057	−0.104 to 0.121	0.882	1.030
SR, Model 1	Physical activity, pandemic (log-transformed, standardised)	−0.003	0.029	−0.061 to 0.055	0.923	1.060
SR, Model 1	Change in physical activity (standardised)	0.014	0.028	−0.042 to 0.070	0.625	1.050
SR, Model 1	Health compared with pre-pandemic	0.010	0.026	−0.040 to 0.061	0.687	1.040
SR, Model 1	Mental health score	−0.070	0.034	−0.137 to −0.002	0.044	1.390
SR, Model 1	Emotional role limitations	0.030	0.028	−0.025 to 0.085	0.286	1.310
SR, Model 1	n = 588; $R^2 = 0.100$; adj. $R^2 = 0.088$; $F(8,579) = 8.34$, $p < 0.001$					
SR, Model 1b (+ self-rated health)	Intercept	2.775	0.197	2.389 to 3.161	< 0.001	
SR, Model 1b (+ self-rated health)	Female gender	0.275	0.063	0.151 to 0.399	< 0.001	1.110

Model	Predictor	B	SE (HC3)	95% CI	p	VIF
SR, Model 1b (+ self-rated health)	Staff cohort	0.103	0.079	−0.052 to 0.259	0.193	1.090
SR, Model 1b (+ self-rated health)	Confirmed COVID-19 infection	−0.011	0.060	−0.130 to 0.107	0.853	1.030
SR, Model 1b (+ self-rated health)	Physical activity, pandemic (log-transformed, standardised)	−0.005	0.031	−0.065 to 0.055	0.872	1.080
SR, Model 1b (+ self-rated health)	Change in physical activity (standardised)	0.010	0.030	−0.048 to 0.068	0.726	1.080
SR, Model 1b (+ self-rated health)	Health compared with pre-pandemic	0.016	0.027	−0.037 to 0.070	0.543	1.070
SR, Model 1b (+ self-rated health)	Mental health score	−0.067	0.037	−0.139 to 0.004	0.065	1.490
SR, Model 1b (+ self-rated health)	Emotional role limitations	0.024	0.030	−0.035 to 0.082	0.427	1.330
SR, Model 1b (+ self-rated health)	Self-rated health, current	−0.019	0.032	−0.081 to 0.043	0.552	1.320
SR, Model 1b (+ self-rated health)	n = 524; R^2 = 0.081; adj. R^2 = 0.065; $F(9,514)$ = 5.19, p < 0.001					
SR, Model 2 (+ EF, EUE)	Intercept	2.682	0.249	2.195 to 3.170	< 0.001	
SR, Model 2 (+ EF, EUE)	Female gender	0.271	0.058	0.157 to 0.384	< 0.001	1.150
SR, Model 2 (+ EF, EUE)	Staff cohort	0.055	0.075	−0.091 to 0.202	0.458	1.130
SR, Model 2 (+ EF, EUE)	Confirmed COVID-19 infection	−0.004	0.055	−0.113 to 0.104	0.935	1.030
SR, Model 2 (+ EF, EUE)	Physical activity, pandemic (log-transformed, standardised)	−0.013	0.029	−0.069 to 0.043	0.643	1.070
SR, Model 2 (+ EF, EUE)	Change in physical activity (standardised)	0.015	0.029	−0.041 to 0.071	0.598	1.050
SR, Model 2 (+ EF, EUE)	Health compared with pre-pandemic	0.017	0.025	−0.033 to 0.066	0.510	1.050
SR, Model 2 (+ EF, EUE)	Mental health score	−0.045	0.033	−0.110 to 0.021	0.179	1.420
SR, Model 2 (+ EF, EUE)	Emotional role limitations	0.027	0.027	−0.027 to 0.081	0.323	1.320
SR, Model 2 (+ EF, EUE)	Enjoyment of Food (EF)	−0.150	0.039	−0.226 to −0.074	< 0.001	1.070
SR, Model 2 (+ EF, EUE)	Emotional Under-Eating (EUE)	0.188	0.035	0.121 to 0.256	< 0.001	1.080
SR, Model 2 (+ EF, EUE)	n = 573; R^2 = 0.194; adj. R^2 = 0.180; $F(10,562)$ = 11.66, p < 0.001					
H, Model 1	Intercept	3.379	0.181	3.025 to 3.733	< 0.001	
H, Model 1	Female gender	0.200	0.060	0.083 to 0.318	< 0.001	1.110
H, Model 1	Staff cohort	−0.109	0.080	−0.267 to 0.048	0.174	1.070
H, Model 1	Confirmed COVID-19 infection	0.166	0.062	0.044 to 0.288	0.008	1.030
H, Model 1	Physical activity, pandemic (log-transformed, standardised)	0.027	0.030	−0.032 to 0.086	0.371	1.060

Model	Predictor	B	SE (HC3)	95% CI	p	VIF
H, Model 1	Change in physical activity (standardised)	−0.012	0.029	−0.069 to 0.044	0.665	1.050
H, Model 1	Health compared with pre-pandemic	0.006	0.026	−0.045 to 0.058	0.809	1.040
H, Model 1	Mental health score	−0.194	0.034	−0.261 to −0.127	< 0.001	1.390
H, Model 1	Emotional role limitations	0.056	0.027	0.003 to 0.110	0.040	1.310
H, Model 1	n = 588; R^2 = 0.159; adj. R^2 = 0.148; $F(8,579)$ = 12.61, p < 0.001					
H, Model 1b (+ self-rated health)	Intercept	3.266	0.203	2.868 to 3.664	< 0.001	
H, Model 1b (+ self-rated health)	Female gender	0.198	0.063	0.074 to 0.323	0.002	1.110
H, Model 1b (+ self-rated health)	Staff cohort	−0.078	0.085	−0.245 to 0.090	0.363	1.090
H, Model 1b (+ self-rated health)	Confirmed COVID-19 infection	0.171	0.065	0.044 to 0.298	0.008	1.030
H, Model 1b (+ self-rated health)	Physical activity, pandemic (log-transformed, standardised)	0.038	0.032	−0.025 to 0.101	0.236	1.080
H, Model 1b (+ self-rated health)	Change in physical activity (standardised)	−0.015	0.031	−0.075 to 0.045	0.621	1.080
H, Model 1b (+ self-rated health)	Health compared with pre-pandemic	0.009	0.028	−0.045 to 0.063	0.744	1.070
H, Model 1b (+ self-rated health)	Mental health score	−0.181	0.038	−0.255 to −0.107	< 0.001	1.490
H, Model 1b (+ self-rated health)	Emotional role limitations	0.072	0.029	0.015 to 0.129	0.014	1.330
H, Model 1b (+ self-rated health)	Self-rated health, current	0.006	0.036	−0.064 to 0.076	0.868	1.320
H, Model 1b (+ self-rated health)	n = 524; R^2 = 0.158; adj. R^2 = 0.144; $F(9,514)$ = 9.79, p < 0.001					
H, Model 2 (+ EF, EUE)	Intercept	1.858	0.242	1.385 to 2.332	< 0.001	
H, Model 2 (+ EF, EUE)	Female gender	0.140	0.055	0.031 to 0.248	0.012	1.150
H, Model 2 (+ EF, EUE)	Staff cohort	0.039	0.074	−0.107 to 0.185	0.600	1.130
H, Model 2 (+ EF, EUE)	Confirmed COVID-19 infection	0.138	0.058	0.026 to 0.251	0.016	1.030
H, Model 2 (+ EF, EUE)	Physical activity, pandemic (log-transformed, standardised)	0.006	0.027	−0.047 to 0.060	0.819	1.070
H, Model 2 (+ EF, EUE)	Change in physical activity (standardised)	−0.004	0.026	−0.055 to 0.046	0.871	1.050
H, Model 2 (+ EF, EUE)	Health compared with pre-pandemic	−0.016	0.025	−0.064 to 0.033	0.525	1.050
H, Model 2 (+ EF, EUE)	Mental health score	−0.179	0.032	−0.242 to −0.116	< 0.001	1.420
H, Model 2 (+ EF, EUE)	Emotional role limitations	0.049	0.027	−0.002 to 0.101	0.062	1.320
H, Model 2 (+ EF, EUE)	Enjoyment of Food (EF)	0.312	0.038	0.238 to 0.386	< 0.001	1.070
H, Model 2 (+ EF, EUE)	Emotional Under-Eating	0.150	0.036	0.078 to 0.221	< 0.001	1.080

Model	Predictor	B	SE (HC3)	95% CI	p	VIF
H, Model 2 (+ EF, EUE)	(EUE)<br>n = 573; R^2 = 0.290; adj. R^2 = 0.277; F(10,562) = 20.75, p < 0.001					

Model	Predictor	B	SE	OR	95% CI (OR)	p
High EOE (≥ 3.5), Model 1b (+ self-rated health)	Intercept	−1.280	0.718	0.278	0.068–1.137	0.075
High EOE (≥ 3.5), Model 1b (+ self-rated health)	Female gender	0.742	0.256	2.099	1.272–3.465	0.004
High EOE (≥ 3.5), Model 1b (+ self-rated health)	Staff cohort	0.430	0.315	1.537	0.829–2.851	0.173
High EOE (≥ 3.5), Model 1b (+ self-rated health)	Confirmed COVID-19 infection	−0.084	0.252	0.919	0.561–1.507	0.738
High EOE (≥ 3.5), Model 1b (+ self-rated health)	Physical activity, pandemic (log-transformed, standardised)	−0.129	0.115	0.879	0.701–1.101	0.262
High EOE (≥ 3.5), Model 1b (+ self-rated health)	Change in physical activity (standardised)	−0.017	0.122	0.983	0.774–1.249	0.891
High EOE (≥ 3.5), Model 1b (+ self-rated health)	Health compared with pre-pandemic	−0.061	0.103	0.940	0.768–1.151	0.551
High EOE (≥ 3.5), Model 1b (+ self-rated health)	Mental health score	−0.192	0.134	0.825	0.635–1.073	0.152
High EOE (≥ 3.5), Model 1b (+ self-rated health)	Emotional role limitations	0.370	0.121	1.447	1.141–1.836	0.002
High EOE (≥ 3.5), Model 1b (+ self-rated health)	Self-rated health, current	−0.165	0.128	0.848	0.659–1.090	0.198
High EOE (≥ 3.5), Model 1b (+ self-rated health)	n = 525; events = 95; Nagelkerke R^2 = 0.152; LR $\chi^2(9)$ = 51.15, p < 0.001					

Note. OLS: ordinary least squares; *B*: unstandardised coefficient; SE (HC3): heteroscedasticity-consistent standard error; CI: confidence interval; VIF: variance inflation factor; OR: odds ratio. Predictors coded as in Tables 9 and 10 of the manuscript (physical activity log-transformed and standardised; change in physical activity standardised; health compared with pre-pandemic 1–5; mental health score 1–6; emotional role limitations 0–3). Model 1b rests on the respondents with a valid current self-rated health (11% structurally missing). AEBQ: Adult Eating Behaviour Questionnaire; EF: Enjoyment of Food; EOE: Emotional Over-Eating; EUE: Emotional Under-Eating; FF: Food Fussiness; FR: Food Responsiveness; SE: Slowness in Eating; H: Hunger; SR: Satiety Responsiveness.

Table S7. Measurement invariance of the eight-factor AEBQ model across gender and across cohort (complete AEBQ data, $n = 621$).

Grouping	Model	χ^2	*df*	CFI	RMSEA	SRMR	ΔCFI	ΔRMSEA	$\Delta\chi^2$ (Δdf)	p ($\Delta\chi^2$)
Gender, ordinal items (WLSMV)	Configural	3049.8	998	0.896	0.082	0.091				
Gender, ordinal items (WLSMV)	Thresholds	3158.4	1066	0.894	0.080	0.091	−0.0021	−0.0019	119.2 (68)	< 0.001
Gender, ordinal items (WLSMV)	Metric (thresholds + loadings)	3206.6	1092	0.893	0.079	0.092	−0.0011	−0.0005	134.7 (26)	< 0.001
Gender, ordinal items (WLSMV)	Scalar (+ intercepts)	3257.3	1118	0.892	0.079	0.092	−0.0012	−0.0005	96.6 (26)	< 0.001
Gender, continuous items (MLR)	Configural	2043.8	998	0.861	0.062	0.079				
Gender, continuous items (MLR)	Metric (loadings)	2101.4	1024	0.856	0.062	0.083	−0.0048	0.0003	57.0 (26)	< 0.001
Gender, continuous items (MLR)	Scalar (+ intercepts)	2179.6	1050	0.850	0.063	0.084	−0.0065	0.0006	81.4 (26)	< 0.001
Cohort, continuous items (MLR)	Configural	2166.2	998	0.854	0.064	0.080				
Cohort, continuous items (MLR)	Metric (loadings)	2187.1	1024	0.853	0.064	0.081	−0.0008	−0.0006	30.6 (26)	0.244
Cohort, continuous items (MLR)	Scalar (+ intercepts)	2245.5	1050	0.849	0.064	0.081	−0.0038	0.0000	58.8 (26)	< 0.001

Note. Multigroup confirmatory factor analysis in lavaan 0.7-2 with semTools 0.5.9 (R 4.5.3). Groups: women $n = 281$, men $n = 340$; students $n = 520$, staff $n = 101$. Ordinal models: WLSMV estimation, delta parameterisation, identification constraints of Wu and Estabrook (2016) via semTools::measEq.syntax; scaled fit indices and scaled χ^2 difference tests. Continuous-item models: robust maximum likelihood (MLR), robust fit indices. The ordinal model could not be fitted across cohort because one response category of one item was unobserved in the staff group. ΔCFI and ΔRMSEA are differences from the preceding model; criteria ΔCFI ≥ −0.010 and ΔRMSEA ≤ 0.015. The single-group WLSMV fit of the combined sample in lavaan (CFI = 0.898, RMSEA = 0.084, scaled statistics) differs from the DWLS fit reported in Table 4 of the manuscript (semopy; CFI = 0.911, RMSEA = 0.057) because of the different test statistic used. CFI: comparative fit index; RMSEA: root mean square error of approximation; SRMR: standardised root mean square residual. AEBQ: Adult Eating Behaviour Questionnaire; EF: Enjoyment of Food; EOE: Emotional Over-Eating; EUE: Emotional Under-Eating; FF: Food Fussiness; FR: Food Responsiveness; SE: Slowness in Eating; H: Hunger; SR: Satiety Responsiveness.

Table S8. Benjamini–Hochberg false discovery rate (FDR) adjustment of the exploratory Spearman correlations: correlations of the AEBQ subscales with physical activity, perceived health and mental health by cohort (upper block, Table 8 of the manuscript) and subscale intercorrelations (lower block, Figure 4 and Table S5).

Cohort	Correlate	EF	EOE	EUE	FF	FR	SE	H	SR
Students	MET-min/week, pandemic	0.057 (0.283)	−0.162 (0.001)	−0.027 (0.636)	−0.071 (0.189)	−0.017 (0.766)	−0.042 (0.440)	−0.106 (0.040)	−0.088 (0.097)
Students	MET-min/week, pre-pandemic	0.078 (0.168)	−0.046 (0.403)	−0.035 (0.530)	−0.060 (0.272)	0.040 (0.480)	−0.070 (0.209)	−0.025 (0.671)	−0.110 (0.037)
Students	Change in MET-min/week (pandemic − pre-pandemic)	−0.060 (0.275)	−0.091 (0.097)	−0.008 (0.906)	0.036 (0.530)	−0.077 (0.169)	0.042 (0.460)	−0.084 (0.138)	0.009 (0.896)
Students	Self-rated health, current	0.107 (0.041)	−0.115 (0.029)	−0.077 (0.168)	−0.057 (0.288)	−0.098 (0.066)	0.000 (0.996)	−0.169 (0.001)	−0.072 (0.189)
Students	Health compared with pre-pandemic	0.068 (0.189)	−0.059 (0.256)	−0.022 (0.671)	0.006 (0.912)	−0.056 (0.272)	0.026 (0.636)	−0.043 (0.403)	−0.013 (0.813)

Cohort	Correlate	EF	EOE	EUE	FF	FR	SE	H	SR
Students	Mental health score	0.056 (0.272)	−0.222 (< 0.001)	−0.153 (0.001)	−0.116 (0.018)	−0.115 (0.019)	0.058 (0.264)	−0.306 (< 0.001)	−0.145 (0.002)
Students	Emotional role limitations	−0.022 (0.671)	0.226 (< 0.001)	0.101 (0.040)	0.022 (0.671)	0.107 (0.029)	−0.014 (0.809)	0.191 (< 0.001)	0.109 (0.029)
Students	Physical limitations	0.048 (0.360)	0.145 (0.002)	0.107 (0.029)	−0.001 (0.990)	0.089 (0.076)	0.037 (0.480)	0.131 (0.006)	0.060 (0.256)
Students	Social interference	−0.034 (0.530)	0.169 (< 0.001)	0.129 (0.007)	0.023 (0.671)	0.063 (0.222)	0.010 (0.878)	0.223 (< 0.001)	0.076 (0.142)
Students	Pain impact	−0.065 (0.209)	0.160 (< 0.001)	0.097 (0.049)	0.139 (0.004)	0.072 (0.168)	−0.003 (0.970)	0.239 (< 0.001)	0.150 (0.002)
Students	Health-perception score	0.156 (0.001)	−0.138 (0.004)	−0.133 (0.006)	−0.150 (0.002)	−0.059 (0.256)	0.047 (0.372)	−0.247 (< 0.001)	−0.157 (0.001)
Staff	MET-min/week, pandemic	−0.205 (0.142)	−0.230 (0.114)	0.068 (0.666)	0.065 (0.666)	−0.022 (0.896)	0.050 (0.735)	−0.063 (0.672)	−0.052 (0.735)
Staff	MET-min/week, pre-pandemic	−0.067 (0.666)	−0.154 (0.295)	0.147 (0.321)	0.001 (0.996)	−0.039 (0.806)	0.209 (0.142)	0.079 (0.617)	0.021 (0.898)
Staff	Change in MET-min/week (pandemic − pre-pandemic)	−0.069 (0.666)	−0.133 (0.374)	−0.222 (0.130)	0.009 (0.970)	−0.036 (0.819)	−0.317 (0.022)	−0.193 (0.162)	−0.220 (0.130)
Staff	Self-rated health, current	0.059 (0.681)	−0.276 (0.044)	0.101 (0.524)	0.007 (0.970)	−0.096 (0.547)	−0.215 (0.130)	−0.180 (0.162)	−0.210 (0.130)
Staff	Health compared with pre-pandemic	0.038 (0.802)	0.085 (0.557)	−0.202 (0.130)	−0.048 (0.735)	−0.085 (0.557)	−0.065 (0.666)	−0.075 (0.617)	−0.183 (0.151)
Staff	Mental health score	0.030 (0.828)	−0.189 (0.151)	−0.229 (0.096)	0.112 (0.429)	−0.316 (0.012)	−0.104 (0.483)	−0.358 (0.005)	−0.217 (0.114)
Staff	Emotional role limitations	0.093 (0.547)	0.218 (0.114)	0.090 (0.549)	−0.185 (0.151)	0.314 (0.012)	0.119 (0.399)	0.323 (0.012)	0.063 (0.666)
Staff	Physical limitations	0.000 (0.996)	0.177 (0.162)	0.132 (0.345)	−0.186 (0.151)	0.187 (0.151)	−0.006 (0.970)	0.266 (0.044)	0.093 (0.547)
Staff	Social interference	−0.012 (0.951)	0.196 (0.142)	0.123 (0.374)	−0.262 (0.044)	0.234 (0.092)	−0.075 (0.617)	0.328 (0.012)	0.043 (0.764)
Staff	Pain impact	0.083 (0.562)	0.234 (0.092)	0.090 (0.549)	−0.125 (0.366)	0.412 (< 0.001)	0.164 (0.194)	0.261 (0.044)	0.170 (0.177)
Staff	Health-perception score	0.031 (0.828)	−0.159 (0.212)	−0.087 (0.557)	−0.165 (0.194)	−0.176 (0.162)	−0.138 (0.312)	−0.196 (0.142)	−0.130 (0.345)

Pair	Students: ρ	Students: q	Staff: ρ	Staff: q	Combined: ρ	Combined: q
EOE–EF	0.144	< 0.001	0.310	0.003	0.138	< 0.001
EUE–EF	−0.059	0.219	−0.113	0.398	−0.061	0.169
EUE–EOE	0.012	0.806	−0.329	0.002	−0.033	0.467
FF–EF	−0.264	< 0.001	−0.189	0.111	−0.244	< 0.001
FF–EOE	0.059	0.219	−0.034	0.807	0.045	0.316
FF–EUE	0.028	0.603	0.100	0.414	0.035	0.449
FR–EF	0.431	< 0.001	0.508	< 0.001	0.442	< 0.001
FR–EOE	0.382	< 0.001	0.376	< 0.001	0.370	< 0.001
FR–EUE	0.138	0.001	0.175	0.130	0.139	< 0.001
FR–FF	−0.148	< 0.001	−0.106	0.398	−0.139	< 0.001
SE–EF	−0.062	0.218	0.085	0.503	−0.045	0.316
SE–EOE	−0.006	0.888	0.078	0.529	0.012	0.745
SE–EUE	0.162	< 0.001	0.105	0.398	0.153	< 0.001
SE–FF	−0.023	0.666	0.023	0.873	−0.018	0.687
SE–FR	−0.022	0.666	0.052	0.684	−0.013	0.745
H–EF	0.281	< 0.001	0.353	< 0.001	0.292	< 0.001
H–EOE	0.443	< 0.001	0.453	< 0.001	0.438	< 0.001
H–EUE	0.230	< 0.001	0.074	0.537	0.208	< 0.001
H–FF	−0.035	0.524	−0.011	0.909	−0.031	0.477
H–FR	0.520	< 0.001	0.523	< 0.001	0.523	< 0.001
H–SE	0.031	0.570	0.143	0.231	0.047	0.315
SR–EF	−0.173	< 0.001	−0.186	0.111	−0.188	< 0.001
SR–EOE	0.151	< 0.001	0.019	0.876	0.140	< 0.001
SR–EUE	0.313	< 0.001	0.308	0.003	0.313	< 0.001

Pair	Students: ρ	Students: q	Staff: ρ	Staff: q	Combined: ρ	Combined: q
SR–FF	0.143	< 0.001	0.221	0.049	0.154	< 0.001
SR–FR	0.014	0.801	−0.250	0.024	−0.029	0.505
SR–SE	0.238	< 0.001	0.222	0.049	0.241	< 0.001
SR–H	0.214	< 0.001	0.167	0.146	0.203	< 0.001

Note. Upper block: Spearman's ρ with the FDR-adjusted q value in parentheses; adjustment applied within each cohort across the 88 tests of the correlate × subscale matrix (11 correlates × 8 subscales). Lower block: Spearman's ρ and q for the 28 subscale pairs, adjusted within each sample (students, staff, combined). Correlations with $q < 0.05$ are considered significant after adjustment; pairwise deletion, n as in Table 8, Figure 4 and Table S5. Variables coded as in Table 8 of the manuscript. MET: metabolic equivalent of task. AEBQ: Adult Eating Behaviour Questionnaire; EF: Enjoyment of Food; EOE: Emotional Over-Eating; EUE: Emotional Under-Eating; FF: Food Fussiness; FR: Food Responsiveness; SE: Slowness in Eating; H: Hunger; SR: Satiety Responsiveness.

Table S9. Sensitivity analyses of the EOE models: equivalence tests with alternative margins (block a), logistic models of high EOE at alternative thresholds (block b) and Model 1 with alternative scoring of the emotional role limitations count (blocks c, OLS, and d, logistic, high EOE ≥ 3.5).

Predictor	B (HC3 SE)	90% CI	TOST p, ±0.05	TOST p, ±0.10	TOST p, ±0.15	TOST p, ±0.20
Change in physical activity (standardised)	−0.037 (0.0352)	−0.095 to 0.021	0.353	0.036	< 0.001	< 0.001
Health compared with pre-pandemic	−0.033 (0.0385)	−0.097 to 0.030	0.334	0.042	0.001	< 0.001
Confirmed COVID-19 infection	0.058 (0.0809)	−0.076 to 0.191	0.538	0.300	0.127	0.040

Predictor	≥ 3.0: OR (95% CI)	≥ 3.0: p	≥ 3.5: OR (95% CI)	≥ 3.5: p	≥ 4.0: OR (95% CI)	≥ 4.0: p
Female gender	1.56 (1.08–2.24)	0.018	2.18 (1.35–3.52)	0.002	1.39 (0.76–2.53)	0.286
Staff cohort	1.48 (0.90–2.41)	0.119	1.42 (0.78–2.59)	0.255	1.23 (0.57–2.68)	0.593
Confirmed COVID-19 infection	1.11 (0.76–1.61)	0.598	0.98 (0.61–1.58)	0.931	1.07 (0.59–1.93)	0.824
Physical activity, pandemic (log, standardised)	0.80 (0.67–0.96)	0.016	0.82 (0.66–1.02)	0.072	0.86 (0.66–1.12)	0.272
Change in physical activity (standardised)	0.97 (0.81–1.17)	0.753	0.96 (0.76–1.21)	0.736	0.97 (0.72–1.30)	0.833
Health compared with pre-pandemic (standardised)	0.97 (0.83–1.15)	0.754	0.90 (0.73–1.09)	0.281	0.82 (0.64–1.05)	0.124
Mental health score	0.81 (0.67–0.98)	0.034	0.76 (0.59–0.96)	0.024	0.63 (0.46–0.86)	0.004
Emotional role limitations	1.33 (1.13–1.58)	< 0.001	1.39 (1.11–1.74)	0.004	1.24 (0.94–1.64)	0.136
n; events; events per predictor	590; 214; 26.8		590; 102; 12.8		590; 57; 7.1	

Predictor	Rule A: B (95% CI)	Rule A: p	Rule B: B (95% CI)	Rule B: p	Rule C: B (95% CI)	Rule C: p
Female gender	0.243 (0.085 to 0.400)	0.003	0.232 (0.069 to 0.396)	0.005	0.241 (0.083 to 0.398)	0.003
Staff cohort	0.198 (−0.007 to 0.403)	0.058	0.194 (−0.023 to 0.411)	0.079	0.187 (−0.017 to 0.391)	0.073
Confirmed COVID-19 infection	0.058 (−0.101 to 0.216)	0.476	0.056 (−0.111 to 0.222)	0.512	0.058 (−0.100 to 0.217)	0.472
Physical activity, pandemic (log, standardised)	−0.083 (−0.161 to −0.005)	0.037	−0.084 (−0.164 to −0.004)	0.039	−0.085 (−0.164 to −0.007)	0.032
Change in physical activity (standardised)	−0.037 (−0.106 to 0.032)	0.298	−0.049 (−0.124 to 0.026)	0.199	−0.035 (−0.105 to 0.034)	0.322

Predictor	Rule A: *B* (95% CI)	Rule A: *p*	Rule B: *B* (95% CI)	Rule B: *p*	Rule C: *B* (95% CI)	Rule C: *p*
Health compared with pre-pandemic	−0.033 (−0.109 to 0.042)	0.386	−0.029 (−0.108 to 0.050)	0.474	−0.033 (−0.108 to 0.043)	0.397
Mental health score	−0.118 (−0.211 to −0.026)	0.012	−0.135 (−0.233 to −0.038)	0.006	−0.114 (−0.207 to −0.020)	0.017
Emotional role limitations	0.129 (0.059 to 0.200)	< 0.001	0.128 (0.055 to 0.200)	< 0.001	0.130 (0.063 to 0.197)	< 0.001

Predictor	Rule A: OR (95% CI)	Rule A: *p*	Rule B: OR (95% CI)	Rule B: *p*	Rule C: OR (95% CI)	Rule C: *p*
Female gender	2.18 (1.35–3.52)	0.002	2.06 (1.25–3.37)	0.004	2.15 (1.33–3.48)	0.002
Staff cohort	1.42 (0.78–2.59)	0.255	1.38 (0.72–2.64)	0.325	1.38 (0.76–2.51)	0.296
Confirmed COVID-19 infection	0.98 (0.61–1.58)	0.931	0.97 (0.59–1.60)	0.909	0.98 (0.61–1.58)	0.933
Physical activity, pandemic (log, standardised)	0.82 (0.66–1.02)	0.072	0.83 (0.66–1.03)	0.094	0.82 (0.66–1.01)	0.063
Change in physical activity (standardised)	0.96 (0.76–1.21)	0.736	0.92 (0.72–1.17)	0.492	0.96 (0.76–1.22)	0.758
Health compared with pre-pandemic	0.90 (0.73–1.09)	0.281	0.88 (0.72–1.09)	0.246	0.90 (0.74–1.10)	0.290
Mental health score	0.76 (0.59–0.96)	0.024	0.73 (0.57–0.94)	0.015	0.77 (0.60–0.98)	0.033
Emotional role limitations	1.39 (1.11–1.74)	0.004	1.38 (1.10–1.74)	0.006	1.41 (1.13–1.76)	0.002

Note. Block a: two one-sided tests (TOST) on the Model 1 coefficients of the three pandemic-related variables (OLS with HC3 standard errors, $n = 590$); equivalence at a given margin (EOE scale points per unit of the predictor) is concluded when TOST $p < 0.05$, i.e. when the 90% confidence interval lies within ±margin; the ±0.10 margin is the primary one. Block b: binary logistic Model 1 with high EOE defined as a score ≥ 3.0, ≥ 3.5 (threshold of Table 10 of the manuscript) or ≥ 4.0; $n = 590$ for all three. Blocks c and d: scoring rules for the three yes/no items on role limitations due to emotional problems — A, unanswered items within an answered block counted as not endorsed (rule used in the manuscript; $n = 590$); B, respondents with any unanswered item excluded ($n = 547$); C, sum prorated to the number of answered items (sum × 3/answered; $n = 590$). Predictors coded as in Tables 9 and 10 of the manuscript. *B*: unstandardised coefficient; CI: confidence interval; OR: odds ratio; SE: standard error. EOE: Emotional Over-Eating.

Table S10. Age and cohort: AEBQ subscale scores of students and staff in the age-overlapping subgroup (block a), association of age group with the EOE, EF, SR and H scores within each cohort (block b) and the adjusted cohort coefficient for EOE in the age-overlapping subgroup (block c).

Subscale	Students aged ≥ 24: mean ± SD (*n*)	Staff aged ≤ 30: mean ± SD (*n*)	U	*p*	*r*
EF	3.59 ± 0.87 (90)	3.83 ± 0.79 (14)	536.5	0.371	0.087
EOE	2.45 ± 0.90 (89)	2.70 ± 1.02 (14)	515.0	0.299	0.102
EUE	2.66 ± 0.84 (89)	2.98 ± 0.79 (14)	493.0	0.209	0.123
FF	2.53 ± 0.73 (88)	2.54 ± 0.43 (14)	610.5	0.961	0.005
FR	2.71 ± 0.61 (89)	3.02 ± 0.85 (14)	452.0	0.098	0.162
SE	2.84 ± 0.91 (88)	2.91 ± 0.74 (14)	594.5	0.838	0.021
H	2.80 ± 0.70 (88)	3.36 ± 0.79 (14)	355.5	0.011	0.251
SR	2.74 ± 0.64 (88)	2.96 ± 0.73 (14)	506.5	0.286	0.105

Cohort	Subscale	Mean score by age group (*n*)	Kruskal–Wallis *H*	*p*	η^2	Spearman ρ (age group)	*p* (ρ)
Students	EOE	18–20: 2.62 (284) / 21–23: 2.42 (215) / 24–26: 2.46 (40) / >26: 2.43 (49)	5.95	0.114	0.005	−0.095	0.021
Students	EF	18–20: 3.80 (283) / 21–23: 3.77 (213) / 24–26: 3.62 (40) / >26: 3.56 (50)	4.42	0.220	0.002	−0.070	0.091

Cohort	Subscale	Mean score by age group (*n*)	Kruskal–Wallis *H*	*p*	η^2	Spearman ρ (age group)	*p* (ρ)
Students	SR	18–20: 2.65 (283) / 21–23: 2.62 (215) / 24–26: 2.68 (40) / >26: 2.80 (48)	2.56	0.464	< 0.001	0.021	0.618
Students	H	18–20: 2.95 (283) / 21–23: 2.85 (214) / 24–26: 2.71 (40) / >26: 2.88 (48)	5.60	0.133	0.004	−0.083	0.046
Staff	EOE	<25: 2.45 (8) / 26–30: 3.03 (6) / 31–40: 2.80 (27) / 41–50: 2.82 (37) / 51–60: 2.86 (18) / 61–70: 2.69 (11) / >70: 2.70 (5)	3.22	0.780	< 0.001	0.025	0.797
Staff	EF	<25: 3.71 (8) / 26–30: 4.00 (6) / 31–40: 3.39 (29) / 41–50: 3.30 (36) / 51–60: 2.90 (17) / 61–70: 3.03 (11) / >70: 3.13 (5)	13.70	0.033	0.073	−0.302	0.001
Staff	SR	<25: 3.25 (8) / 26–30: 2.58 (6) / 31–40: 2.77 (27) / 41–50: 2.81 (37) / 51–60: 2.91 (18) / 61–70: 2.66 (11) / >70: 2.95 (5)	4.39	0.624	< 0.001	−0.027	0.781
Staff	H	<25: 3.33 (8) / 26–30: 3.40 (6) / 31–40: 2.75 (27) / 41–50: 2.75 (37) / 51–60: 2.82 (18) / 61–70: 2.51 (11) / >70: 2.55 (5)	12.45	0.053	0.061	−0.221	0.019

Outcome	Model	Staff cohort: *B* (95% CI)	*p*	*n*
EOE	Model 1 of the manuscript refitted in the age-overlapping subgroup	0.238 (−0.365 to 0.841)	0.439	87

Note. Block a: students in the age groups 24–26 and > 26 years ($n = 100$) and staff in the age groups < 25 and 26–30 years ($n = 15$); Mann–Whitney *U* test, $r = Z/\sqrt{N}$, Bonferroni-corrected threshold for eight subscales $p < 0.00625$; *n* with a valid score in parentheses. Block b: Kruskal–Wallis *H* test across the age groups of each cohort ($\eta^2 = (H - k + 1)/(n - k)$) and Spearman's ρ between the ordered age group and the subscale score. Age-group categories differ between the two questionnaires (students: 18–20, 21–23, 24–26, > 26; staff: < 25, 26–30, 31–40, 41–50, 51–60, 61–70, > 70). Block c: ordinary least squares with HC3 standard errors, predictors as in Table 9 of the manuscript, respondents of the age-overlapping subgroup with complete data; *B*: unstandardised coefficient; CI: confidence interval. AEBQ: Adult Eating Behaviour Questionnaire; EF: Enjoyment of Food; EOE: Emotional Over-Eating; EUE: Emotional Under-Eating; FF: Food Fussiness; FR: Food Responsiveness; SE: Slowness in Eating; H: Hunger; SR: Satiety Responsiveness.

Figure S1. Emotional Over-Eating score against moderate-to-vigorous physical activity during the pandemic, by cohort.

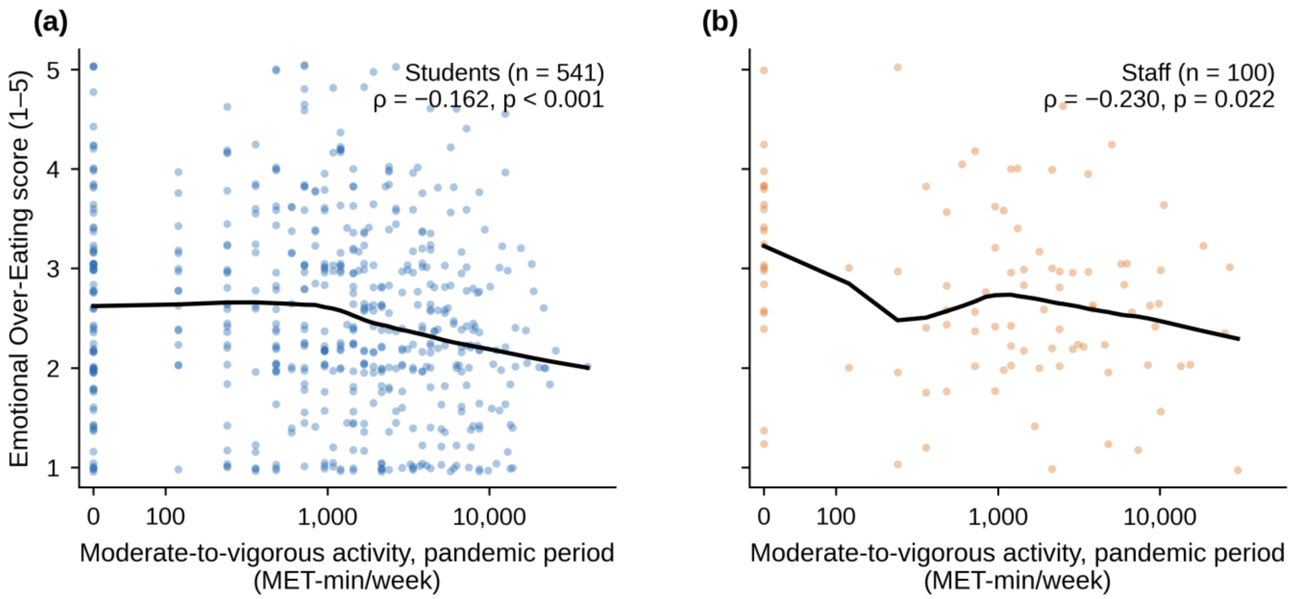

(a) Students (n = 541); (b) staff (n = 100). Each point is one respondent (vertical jitter of ± 0.05 added for visibility); the black line is a LOWESS smoother (span 0.5); Spearman's ρ and unadjusted p are given in each panel (exploratory comparison; FDR-adjusted q values in Table S8). Physical activity in MET-min/week (GPAQ scoring) on a symmetric logarithmic axis (linear below 100 MET-min/week, logarithmic above), so that zero values are shown at zero. Blue: students; orange: staff. EOE: Emotional Over-Eating; GPAQ: Global Physical Activity Questionnaire; LOWESS: locally weighted scatterplot smoothing; MET: metabolic equivalent of task.
